# Re‐analysis of single‐cell transcriptomics reveals a critical role of TNS1 gene in driving contractile VSMC transdifferentiation into macrophage‐like SMC and atherosclerotic plaque instability

**DOI:** 10.1002/ctm2.70664

**Published:** 2026-04-20

**Authors:** Shuang Yang, Rui Fu, Xiaoxiao Ren, Mengyi Sun, Zhifan Li, Shufeng Chen, Bin Yang, Na Shi, Jue Ye, Chenyang Shen, Xianqiang Wang, Yongchun Cui, Naqiong Wu, Xiangfeng Lu, Dongfeng Gu, Laiyuan Wang

**Affiliations:** ^1^ Key Laboratory of Cardiovascular Epidemiology & Department of Epidemiology, State Key Laboratory of Cardiovascular Disease, Fuwai Hospital, National Center for Cardiovascular Diseases Chinese Academy of Medical Sciences and Peking Union Medical College Beijing China; ^2^ Department of Cardio‐Metabolic Medicine Center, Fuwai Hospital, National Center for Cardiovascular Diseases Chinese Academy of Medical Sciences and Peking Union Medical College Beijing China; ^3^ State Key Laboratory of Cardiovascular Disease, Fuwai Hospital, National Center for Cardiovascular Diseases Chinese Academy of Medical Sciences and Peking Union Medical College Beijing China; ^4^ Department of Vascular Surgery, State Key Laboratory of Cardiovascular Disease, Fuwai Hospital, National Center for Cardiovascular Diseases Chinese Academy of Medical Sciences and Peking Union Medical College Beijing China; ^5^ Department of Surgery, Fuwai Hospital, National Center for Cardiovascular Diseases Chinese Academy of Medical Sciences and Peking Union Medical College Beijing China; ^6^ Beijing Key Laboratory of Preclinical Research and Evaluation for Cardiovascular Implant Materials, Animal Experimental Center, State Key Laboratory of Cardiovascular Disease, National Center for Cardiovascular Diseases Fuwai Hospital Chinese Academy of Medical Sciences & Peking Union Medical College Beijing China; ^7^ School of Public Health and Emergency Management, School of Medicine Southern University of Science and Technology Shenzhen China

**Keywords:** atherosclerotic plaque instability, macrophage (MP)‐like SMC, single‐cell sequencing (scRNA‐seq) dataset, tensin1 (TNS1) gene, VSMC transdifferentiation

## Abstract

**Background:**

Vascular smooth muscle cell (VSMC) phenotype switching plays a significant role in the pathogenesis of atherosclerosis (AS). However, the subtypes of VSMC transdifferentiation and their impact on AS progression and atherosclerotic plaque instability remains unclear.

**Methods:**

We reanalysed scRNA‐seq datasets of GSE155513 and GSE253903 and performed single‐sample gene set enrichment analysis (ssGSEA) in three transcriptome datasets from unstable plaques to determine the major subtypes contributing the most to plaque instability. Using high‐dimensional weighted gene co‐expression network analysis (hdWGCNA), we identified hub genes in macrophage (MP)‐like smooth muscle cells (SMCs) of unstable plaques. We conducted cell communication analysis according to tensin1 (*TNS1*) gene levels in VSMCs. *TNS1* expression was analysed in human AS plaques. Finally, an AS model was established in VSMC‐specific *Tns1* knockout *ApoE* ^−/−^ mice to validate the causative role of TNS1 on atherosclerotic lesions.

**Results:**

MP‐like SMC was identified as the key subtype for plaque instability. hdWGCNA analysis for MP‐like SMC identified blue module as the key gene module involved in unstable plaques. Decreased *TNS1* expression in VSMCs was positively correlated with the down‐regulation of contractile VSMC marker genes, *SRF* and *MYCOD* genes, negatively correlated with the up‐regulation of *CD68* and *KLF4* genes, and activated VCAM, PDGF, THBS and CXCL signalling pathways. *TNS1* mRNA expression levels were lower in human atherosclerotic arteries than in healthy arteries, and even lower in unstable plaques than in early and stable plaques. TNS1 protein levels in VSMCs were lower in human atherosclerotic plaques than in healthy arteries, and even lower in advanced plaques than in early plaques. VSMC‐specific *Tns1* gene deficiency aggravated AS progression and enhanced plaque instability with increased MP‐like SMC transdifferentiation.

**Conclusion:**

The reduction of *TNS1* gene in VSMCs might drive contractile VSMC transdifferentiation into MP‐like SMC, the major subtype contributing to plaque instability. In vivo experimental results confirmed the role of *Tns1* gene in contractile VSMC transdifferentiation into MP‐like SMC and plaque instability.

## Highlights

- Macrophage (MP)‐like SMC was identified as the major SMC subtype contributing the most to atherosclerotic progression and plaque instability.
- The downregulation of *TNS1* gene in VSMCs might drive contractile VSMC transdifferentiation into MP‐like SMC and atherosclerotic plaque instability.
- VSMC‐specific *Tns1* gene deficiency aggravated AS progression and enhanced atherosclerotic plaque instability with intensified MP‐like SMC transdifferentiation.

## Graphical abstract

• Macrophage (MP)‐like smooth muscle cell (SMC) contributed the most to plaque instability.

• *TNS1* was identified as a hub gene driving contractile vascular smooth muscle cell (VSMC) transdifferentiation into MP‐like SMC, in addition to initiating VSMC phenotyping switching.

• ZEB1 might function as a transcription factor for *TNS1* gene in VSMCs.

• VSMC‐specific *Tns1* gene deficiency aggravated atherosclerosis (AS) progression, enhanced atherosclerotic plaque instability and drived MP‐like SMC transdifferentiation.

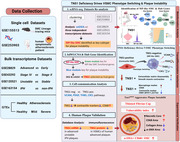

## 1 INTRODUCTION

Atherosclerosis (AS) constitutes the primary pathological underpinning for the majority of atherosclerotic cardiovascular diseases (ASCVD).^1^ Clinical prognosis of destructive events, such as myocardial infarction and stroke, is largely determined by the instability of atherosclerotic plaques.^2^, ^3^ Vulnerable plaques are characterised by a thin fibrous cap, a large necrotic core, intense inflammation within the plaque, intraplaque haemorrhage (IPH), a high risk of rupture and a paucity of vascular smooth muscle cells (VSMCs)—the key cells responsible for maintaining fibrous cap integrity.^2^ Rupture of such plaques triggers acute thrombotic event.^2^ Therefore, identifying the characteristics of unstable atherosclerotic plaques as well as novel pathogenic genes and revealing the new pathological and physiological mechanisms, is of great significance for improving the prevention and treatment levels of ASCVD.

Traditionally regarded as stable and contractile cells in the media layer of arteries.^4^ VSMCs are now considered to be the key players in AS progression due to their remarkable phenotypic plasticity.^5^ In response to atherogenic stimuli, VSMCs can undergo phenotype switching, losing their contractile markers and acquiring multiple functions.^6^ The traditional view holds that foam cells originate from the differentiation of monocytes into macrophages (MPs). However, lineage‐tracing studies have confirmed that foam cells also originate from endothelial cells and VSMCs, and more than half of the CD68^+^ MPs are derived from VSMCs.^7^, ^8^, ^9^ In human coronary atherosclerotic plaques, at least 50% of foam cells are originating from VSMCs, and approximately 40% of MPs are originating from VSMCs, rather than from a monocyte origin.^10^ In ApoE^−/−^ mouse model, 60%–70% of foam cells within plaques are derived from VSMCs.^11^ Recently, single‐cell RNA sequencing (scRNA‐seq) and VSMC‐lineage‐tracing mice revealed unexpected heterogeneity of the cell sources of VSMCs in atherosclerotic lesions,^5^, ^12^, ^13^ indicating that SMCs could undergo clonal expansion and differentiate into multiple phenotypes, such as fibroblasts (FBs)‐like SMCs, mesenchymal‐like SMCs, chondrocyte‐like SMCs, osteogenic SMCs, foamy SMCs and MP‐like SMCs.^14^, ^15^ Some studies have revealed that MP‐like SMCs are associated with the driving of plaque instability,^16^, ^17^ and MP‐like SMCs‐originating foam cells secrete matrix metalloproteinases (MMPs) to degrade ECM in the fibrous cap.^4^ These cells down‐regulate the classical contractile SMC markers (ACTA2, CNN1 and MYH11) and acquire MP‐related marker (CD68) and partial functions,^5^, ^18^ such as phagocytosis and lipid uptake, thereby forming SMC‐derived foam cells. However, it remains controversial which SMC subtype—FB‐like SMC, MP‐like SMC or other SMC subtype—plays the leading pro‐atherogenic role in unstable atherosclerotic plaques. Thus, identifying the key SMC subtype contributing greatly to unstable atherosclerotic plaques and elucidating the underlying mechanisms through which contractile VSMCs transdifferentiate is of great significance for understanding and treating rupture of unstable atherosclerotic plaques. To solve these uncertainties, we reanalysed scRNA‐seq datasets deriving from AS models of SMC‐lineage‐tracing mice and symptomatic human AS patients, founding that the MP‐like SMCs played a major role in late‐stage and unstable atherosclerotic plaques. High‐dimensional weighted gene co‐expression network analysis (hdWGCNA) analysis identified two important gene modules in MP‐like SMC, representing the expression characteristics of MP‐like SMCs in unstable atherosclerotic plaques. Analysis of the expression of these two modules in contractile VSMCs and MP‐like SMCs of unstable atherosclerotic plaques indicated that blue module genes might play an important role in contractile VSMC transdifferentiation into MP‐like SMC and unstable atherosclerotic plaques. Subsequent cross analysis of blue modules with differentially expressed genes of MP‐like SMCs in scRNA‐seq dataset GSE253903, least absolute shrinkage and selection operator (LASSO) regression analysis in bulk RNA‐seq dataset GSE12052 combined with pseudotime trajectory analysis in scRNA‐seq dataset GSE155513 demonstrated that the decreased expression of Tensin‐1 (*TNS1*) gene in VSMCs might drive contractile VSMC transdifferentiation into MP‐like SMC.

TNS1 is a cytoplasmic phosphoprotein that localises to focal and fibrillar adhesions, linking the extracellular matrix to the cytoskeleton.^19^ As a scaffold protein with multiple domains, it governs extracellular‑to‑intracellular and intracellular‑to‑extracellular signal transduction. These signalling events are critical for a wide range of biological functions. TNS1 has been implicated in valve morphogenesis^20^ and is recently reported to be a critical checkpoint protein for keeping the contractile phenotype of VSMCs.^21^ Its role in arterial pathologies is emerging, for instance, in abdominal aortic aneurysm, miR‐3154‐mediated suppression of TNS1 drived contractile VSMCs towards a destructive synthetic state by disrupting integrin β1 signalling.^22^ Nevertheless, the role of *TNS1* gene in AS progression and plaque instability remains largely unknown.

Therefore, we performed correction of the expression of *TNS1* gene in VSMCs with contractile VSMC marker genes, MP marker gene and corresponding transcription factor (TF) genes critical for VSMC phenotype switching. Lower expression of *TNS1* gene have decreased expression of contractile VSMC marker genes, *SRF* and *MYCOD* gene, increased expression of MP marker gene *CD68* and *KLF4* gene, inflammatory factor genes (*CCL2*, *CXCL12* and *VCAM‐1*) and *MMP2*. Then, we also conducted cell communication analysis in *TNS1*‐low or *TNS1*‐high VSMCs and found that the expression levels of *TNS1* gene in VSMCs was related to important pathways in progression of AS. Finally, in vivo experimental validation demonstrated that VSMC‐specific *Tns1* gene deficiency aggravated atherosclerotic plaque instability with an increased MP‐like SMC transdifferentiation.

## 2 METHOD

### 2.1 Data collection

We obtained all data from two public repositories: GEO database (http://www.ncbi.nlm.nih.gov/geo) and GTEx Portal (www.gtexportal.org/). Mouse scRNA‐seq dataset (GSE155513) was derived from arterial tissues of SMC‐lineage‐tracing mouse AS models, including Ldlr^−/−^ mice at 0, 8, 16, 26 weeks and ApoE^−/−^ mice at 8, 16, 22 weeks of HFD feeding, and human scRNA‐seq dataset GSE253903 was derived from carotid atherosclerotic plaques from 12 subjects (six symptomatic and six asymptomatic). GSE43292 microarray data were derived from carotid endarterectomy specimens obtained from 32 patients. For each patient, two samples were collected: one from the atheromatous plaque (Stary grade ≥IV, including the core and shoulder regions) and one from macroscopically intact tissue at a distant site (Stary grades I–II). Microarray gene expression data of GSE28829 dataset included plaques from patients with early AS (*n* = 13) and advanced AS (*n* = 16). Bulk RNA‐seq data of GSE120521 dataset were obtained from four patients with plaques, and stable and unstable regions were dissected, with unstable regions characterised by visible areas of plaque rupture. Microarray gene expression data of GSE163154 dataset included 27 tissue samples from patients with IPH and 16 tissue samples from patients without IPH. Microarray gene expression data of GSE100927 dataset included control tissue (*n* = 35) and atherosclerotic plaque (*n* = 69). Detailed information of the selected datasets is presented in Table S1.

### 2.2 GTEx database re‐analysis

The aortic expression data were downloaded from the GTEx database. Based on the pathological annotations of the samples, we divided the samples into normal and atherosclerotic samples, and excluded some samples that contained veins, adipose tissue and nerves. According to the pathological annotations, we further classified the samples into healthy, mild and severe categories.^23^ Detailed information of the selected datasets is presented in Table S1. The Wilcoxon rank‐sum test was applied for statistical comparisons between groups to account for the non‐normal distribution of TPM values. *p* < .05 was considered statistically significant.

### 2.3 GEO microarray dataset re‐analysis

For public microarray dataset validation (GSE100927, GSE43292, GSE28829, GSE163154, GSE47744), the normalised log2‐transformed expression values of *TNS1* or *ZEB1* were extracted. We assessed statistical significance using an unpaired two‐tailed Welch's *t*‐test, a method that does not assume homogeneity of variances across groups.

### 2.4 GEO bulk RNA‐seq dataset

For public bulk RNA‐seq dataset validation (GSE120521), FPKM values were normalised using a **log2(FPKM + 1)** adjustment to linearise the dynamic range and approximate a normal distribution. We assessed statistical significance using an unpaired two‐tailed Welch's *t*‐test, a method that does not assume homogeneity of variances across groups.

### 2.5 Analysis of GSE155513

To harmonise scRNA‐seq data from *ApoE* ^−/−^ and *Ldlr^−/−^* mice and experimental timepoints in the GSE155513 dataset, we employed the Seurat R package (version 5). We choose the ZsGreen1^+^ cells (SMC‐lineage cells) to analysis. Low‑quality cells were removed through an initial quality control step. Only cells with more than 200 detected genes (nFeature_RNA >200) and a mitochondrial transcript proportion below 30% (percent.mt <30) were kept for subsequent analyses. After preliminary data processing, we obtained an expression matrix containing 25 422 cells. We next normalised the filtered expression matrix using the LogNormalise approach with a scaling factor of 10 000.

To effectively remove technical batch effects while preserving biological variation, we utilised the canonical correlation analysis (CCA) integration pipeline. The dataset was split by genetic background (*ApoE* ^−/−^ vs. *Ldlr^−/−^* mice), and the top 1500 highly variable genes (HVGs) were independently identified for each subset using the vst selection method. Integration anchors were identified using the FindIntegrationAnchors function with the CCA reduction method based on the first 20 dimensions (dims = 1:20). Subsequently, a harmonised expression matrix was generated using the IntegrateData function.

We began by scaling the integrated data and then conducting principal component analysis (PCA). For non‑linear dimensionality reduction, we used the first 20 principal components to build a uniform manifold approximation and projection (UMAP) representation. Cells were grouped by applying a shared nearest neighbour (SNN) algorithm that optimises modularity, using a resolution parameter of .5.

For the accurate identification of marker genes and visualisation (DotPlots), the default assay was switched back to the unintegrated ‘RNA’ layer to reflect true biological expression counts. Dotplot showed expression of marker genes for each SMC subtype. Marker gene^14^, ^24^, ^25^, ^26^: Contractile SMC(SMC) (*Myh11*, *Acta2* and *Tagln*), MP‐like SMC (*Cd68*, *Lgals3*, *Apoe* and *Lpl*), mesenchymal‐like SMC (*Ly6a* and *Vcam1*), FB‐like SMC (*Fn1*, *Lum*, *Dcn* and *Bgn*). We identified cluster‑specific marker genes with the FindAllMarkers function, which applies the Wilcoxon rank‑sum test. Only genes with an adjusted *p* value <.05 and an avg_log2FC > .25 were retained.

### 2.6 Analysis of GSE253903

We used Seurat (v5) to analyse the UMI count matrix. First, we discarded any gene found in fewer than three cells. Next, we kept only cells that had between 200 and 4000 detected genes and less than 3% mitochondrial reads. This left 53 101 cells. The 2000 most variable genes were then identified, and data scaling was subsequently performed. PCA was carried out on those genes, then we took the first 18 principal components (FindNeighbours). Clusters were called with FindClusters at resolution .7. For visualisation, we ran UMAP using the top 10 PCs. To find cluster‐specific markers, we applied the Wilcoxon test (FindAllMarkers) with adjusted *p* < .05 and avg_log2FC > .25. Finally, we annotated this cell manually based on known marker genes. Marker gene^27^: DC (*CLEC4C*), B cell (*CD79A*), VSMC (*MYH11*), endothelial cell (*CHD5*), mononuclear/MP (*C1QB*), neutrophil (*S100A8*), FB (*DCN*), Mast cell (*TPSB2*), natural killer cell (*NKG7*), T cell (*IL7R*).

### 2.7 SMC sub‐cluster analysis (GSE253903)

After annotation of the main cell populations, SMCs were extracted and re‐clustered. From this subpopulation, 2000 variable genes were selected for PCA, which captured variation specific to SMCs. Scaling was performed based on the entire SMC gene set. The first 15 principal components were determined to be significant and used for subsequent steps. With the resolution parameter set to .4, we performed cell clustering using the Seurat Findclusters function. Dotplot showed the expression of marker genes for each SMC subtype. Marker gene^14^, ^24^, ^25^, ^26^: SMC (*MYH11*, *ACTA2* and *TAGLN*), MP‐like SMC (*PTPRC*, *CD68* and *CD74*), mesenchymal‐like SMC (*VCAM1* and *PDGFRA*), FB‐like SMC (*FN1*, *LUM*, *DCN* and *BGN*), chondromyocyte SMC (C*YTL1*), osteogenic SMC (*DLX5*).

### 2.8 Pseudotime trajectory analysis

Monocle3 is a trajectory inference tool designed for reconstructing cellular dynamics from scRNA‐seq data. It models temporal transitions in cell states, inferring potential developmental trajectories or state transition pathways using an unsupervised learning framework. By leveraging transcriptomic similarity, Monocle3 constructs a topological graph representing cellular states. In this study, Monocle3 (v1.3.5) was applied along with UMAP for dimensionality reduction and trajectory reconstruction. Based on biological relevance, we selected the SMC cluster that exhibited the highest level of contractile VSMC marker genes as the root node.

### 2.9 Single‐sample gene set enrichment analysis

From the scRNA‐seq analysis of atherosclerotic samples, cluster‑specific marker genes were derived for each SMC subtype using the criteria avg_log2FC > .15 and adjusted *p* value <.05. These gene signatures were subsequently applied in single‐sample gene set enrichment analysis (ssGSEA) via the GSVA package to estimate the relative abundance of each SMC subtype within three bulk transcriptomic datasets derived from human vulnerable plaques. The resulting signature scores were visualised using box plots generated with the ggplot2 package.

### 2.10 High‐dimensional weighted gene co‐expression network analysis for MP‐like SMC

To identify MP‐like SMC‐associated hub genes in symptomatic AS patients, we applied hdWGCNA^28^ to human scRNA‑seq data. When the soft threshold was 9, there were a total of 22 modules. Module–trait relationship analysis revealed modules significantly associated with symptomatic AS. Hub genes were defined by intra‑module connectivity within these modules, with the top 65 considered key genes for symptomatic AS.

### 2.11 Functional enrichment analysis

We employed the clusterProfiler package (v4.4) in R to perform Gene Ontology (GO) and KEGG enrichment analyses on the target genes, aiming to uncover their potential biological roles and mechanisms. All gene sets were sourced from the MSigDB (https://www.gsea‐msigdb.org/gsea/downloads.jsp).

### 2.12 Identification of differentially expressed genes

Differentially expressed genes (DEGs) were identified with FindAllMarkers using the following thresholds with adjusted *p* value <.05 and avglog2FC > .5, detection in at least 25% of cells within the cluster of interest (pct.1 > .25), and a minimum difference in detection rate of 20% compared to other clusters (pct.1–pct.2 > .20).

### 2.13 The analysis of DEGs in *TNS1*‐postive and *TNS1*‐negative groups

To evaluate the expression patterns of specific markers within VSMC subpopulation, we performed differential expression analysis between defined sub‐clusters (*TNS1*‐pos vs. *TNS1*‐neg). The scRNA‐seq data were normalised using the CellChat framework (v1.6.1). The log_2_FC was calculated based on the difference in mean log‐normalised expression values, consistent with the standard Seurat‐based heuristic: **log_2_FC = log_2_(mean(expm1(*X*_target_)) + 1) − log_2_(mean(expm1(*X*_control_)) + 1)**, where *X* represents the normalised counts. The two‑sided Wilcoxon rank‑sum test was used to assess statistical significance, a non‐parametric approach robust to the zero‐inflated nature of scRNA‐seq data.

Visualisation was performed using ‘Raincloud plots’ to simultaneously display the probability density (via half‐violin plots), individual cellular expression levels (via jittered raw data points) and summary statistics (via box‐and‐whisker plots), providing a comprehensive view of the transcriptional heterogeneity.

### 2.14 Correlation analysis between *TNS1* expression in VSMCs and pathway signatures (ssGSEA and correlation visualisation)

To quantitatively evaluate the relationship between *TNS1* expression and specific biological pathway activities at the single‐cell resolution, pathway enrichment scores were calculated using ssGSEA. Subsequently, a PCA was performed to evaluate the linear association of the normalised expression levels of *TNS1* with the ssGSEA enrichment scores of the selected Gene Ontology Biological Processes (GOBP).

To effectively visualise these correlations and overcome the visual overplotting artefacts inherent in large‐scale scRNA‐seq datasets, point density scatter plots were generated using the ggpointdensity package in R. Individual cells were mapped to a 2D local density gradient using a viridis colour palette (plasma option). A linear regression trendline with a 95% confidence interval was fitted (lm method) to illustrate the overall correlation trajectory.

### 2.15 Functional gene signature scoring

To evaluate the overall functional states of VSMC subpopulations, we performed module scoring using the AddModuleScore function in Seurat. Two distinct gene signatures were curated based on canonical markers: a contractile signature (comprising *ACTA2*, *MYH11*, *CNN1*, *TAGLN*, *MYL9*, *LMOD1* and *SYNPO2*) and an inflammatory signature (comprising *CCL2*, *VCAM1*, *IL6*, *CXCL12*, *LGALS3*, *SPP1* and *MMP2*). The module score for each cell was calculated by subtracting the average expression of a bin‐matched control gene set (*n* = 100) from the average expression of the target signature to account for technical variation and sequencing depth. The Mean ΔScore was defined as the difference in average module scores between *TNS1*‐neg and *TNS1*‐pos groups. The two‐sided Wilcoxon rank‐sum test was employed to determine statistical significance.

### 2.16 Cell–cell communication analysis

Communication between VSMC was quantified using CellChat^29^ (version 1.6.1). Comparisons between groups were performed by stratifying cells based on *TNS1* expression status (positive vs. negative). The total number of interactions and alterations in communication patterns between specific cell types were assessed across groups. Differences in signalling pathways were identified by comparing the overall information flow between the two groups. Furthermore, significant ligand–receptor interactions were visualised as bubble plots using the netVisual_bubble function, with the remove.isolate parameter set to TRUE to exclude isolated interactions.

### 2.17 VSMC‐specific context transcription factor regulatory network inference and analysis

TF regulatory network analysis was performed on VSMCs from GSE253903 dataset using hdWGCNA R package. The workflow comprised five main stages: TF motif scanning, gene set selection for TF network analysis, XGBoost‐based modelling and definition of TF regulons.

#### 2.17.1 TF motif scanning

Position weight matrices (PWMs) for vertebrate TFs were obtained from the JASPAR2020 CORE collection using TFBSTools. The MotifScan function was applied to identify TF binding motifs within promoter regions (defined as ± 2 kb from the transcription start site) of all genes, using the human genome assembly hg38 (BSgenome.Hsapiens.UCSC.hg38) and gene annotation from EnsDb.Hsapiens.v86. This generated a binary matrix indicating the presence of each TF motif in the promoter of each gene.

#### 2.17.2 Gene set selection for TF network analysis

For TF regulatory network construction, all genes encoding TFs (based on motif information) were selected together with all genes assigned to non‑grey co‑expression modules from the hdWGCNA analysis. Genes in the grey module (representing unassigned or noisy genes) were excluded. The expression matrix for these selected genes was prepared with VSMCs as the target group.

#### 2.17.3 TF regulatory network construction using XGBoost

Directed TF regulatory networks were constructed using an extreme gradient boosting (XGBoost) approach. For each target gene, an XGBoost model was trained to predict its expression based on the expression of all TFs whose motifs were present in the gene's promoter. Model parameters were set as follows: objective = ‘reg:squarederror’, max_depth = 1, eta = .1, alpha = .5. For each TF–target pair, a Gain score (representing the improvement in model accuracy contributed by that TF, reflecting regulatory importance) and the Pearson correlation coefficient (Cor) were calculated.

#### 2.17.4 Definition of TF regulons

The raw TF network was refined to retain high‐confidence regulatory relationships using strategy A, which selects the top TFs for each target gene based on regulatory importance. Specifically, for each gene, top 10 TFs with the highest Gain values were retained, with a minimum regulatory score threshold of .01. This generated a set of regulons—each defined as the collection of high‐confidence target genes for a given TF.

#### 2.17.5 Identification and visualisation of key TFs

To identify globally important TFs in VSMCs, all TFs were ranked by two metrics: the number of unique target genes assigned to each TF (regulatory breadth) and the total cumulative Gain across all targets of each TF (regulatory impact). Bar plots were generated using ggplot2. For gene‐centric analysis, TFs predicted to regulate the gene of interest (e.g., *TNS1*) were extracted from both the raw TF network and the refined regulon list. The top TFs were visualised using bar plots coloured by correlation coefficient to indicate activating (positive Cor) or repressive (negative Cor) relationships. Directed regulatory networks centred on the target gene were constructed using the igraph package and visualised with ggraph, with edge width proportional to Gain and edge colour representing correlation direction. For TF‐centric analysis, the regulon of a specific TF (e.g., ZEB1) was visualised using RegulonBarPlot with a Gain cutoff of .2, displaying the top target genes ranked by Gain and coloured by correlation.

### 2.18 Prediction of TFs and potential binding sites for *TNS1* gene

To identify the TFs regulating *TNS1* gene, we initially employed the online bioinformatic platforms animal TFDB (https://guolab.wchscu.cn/AnimalTFDB4) and ChEA3(https://maayanlab.cloud/chea3) to screen for potential TFs of *TNS1* gene. To further characterise the regulatory mechanism, the JASPAR(https://jaspar.elixir.no) database was utilised to predict the specific TF binding sites within the promoter sequences of *TNS1* gene based on high‐quality PWMs.

### 2.19 Cell culture and transfection

The following culture condition was used: (1) 37°C, 5% CO_2_ incubator; (2) mycoplasma‑free status verified by routine testing. Human aortic smooth muscle cells (HASMCs) were seeded in 12‑well plates (1.0 × 10^5^ cells/well), cultured for 48 h and transfected with 50 nM of the indicated siRNAs (si‐*TNS1*‐homo:5′‐GCCUGUAUGCUAAGGUGAAdTdT‐3′/5′‐UUCACCUUAGCAUACAGGCdTdT‐3′) using LipofectamineTM RNAiMax (Invitrogen).

### 2.20 Western blotting

Cells were incubated in RIPA buffer supplemented with complete protease inhibitor (Roche) on ice for 30 min. Following centrifugation (12 000 rpm, 5 min, 4°C), the supernatants were recovered. Protein samples were normalised to equal amounts and subjected to electrophoresis using either 10% SDS‑polyacrylamide gels or 4%–12% Bis–Tris gels (GenScript). We then electroblotted the proteins onto nitrocellulose membranes. Primary antibodies used in this study were: anti‑TNS1 (1:700, ab233133, Abcam), anti‑β‑tubulin (1:1000, 10094‑1‑AP, Proteintech) and anti‑LGALS3 (1:700, 82024‑1‑RR, Proteintech).

### 2.21 Human tissue samples

We obtained carotid atherosclerotic plaques from patients undergoing carotid endarterectomy and internal mammary artery specimens from Fuwai Hospital. The Ethics Committee of Fuwai Hospital approved this study (No. 2022‐1780). In compliance with the Declaration of Helsinki, we secured written informed consent from every participant or their next of kin. Clinical information is detailed in Table S2.

### 2.22 Animals

Animal procedures were approved by Fuwai Hospital's Institutional Animal Care and Use Committee (FW‑2022‑0054) and adhered to the NIH Guide for the Care and Use of Laboratory Animals. A CRISPR/Cas9‑based method was employed to generate VSMC‑specific Tns1‑knockout mice (Beijing Biocytogen Co., Ltd.). Briefly, the CRISPR design tool was used to design sgRNAs (http://crispr.mit.edu). VSMC‐specific *Tns1*‐knockout (*Tns1* ^flox/flox^/Cre+;*Tns1* ^SMCKO^) mice were generated by crossbreeding *Tns1* ^flox/flox^ mice with SM22α‐Cre transgenic mice. *Tns1* ^flox/flox^ *ApoE* ^−/−^ mice were generated by crossbreeding *Tns1* ^flox/flox^ mice with *ApoE* ^−/−^ mice. Tagln‐Cre/+*ApoE* ^−/−^ mice were generated by crossbreeding SM22α‐Cre transgenic mice with *ApoE* ^−/−^ mice. VSMC‐specific *Tns1*‐knockout mice under the background of *ApoE* ^−/−^ (*Tns1* ^SMCKO^ *ApoE* ^−/−^) mice were generated by crossbreeding *Tns1* ^flox/flox^ *ApoE* ^−/−^ mice with Tagln‐Cre/+*ApoE* ^−/−^ mice. Littermate *Tns1* ^flox/flox^ *ApoE* ^−/−^ mice were used as controls. Genotypes were confirmed by PCR with genotyping assay kit mouse tail (Beyotime). The primers used for genotyping were listed in Table S5. Eight weeks old male *Tns1* ^SMCKO^ *ApoE^−/−^* and *Tns1* ^flox/flox^ *ApoE^−/−^* mice were assigned to high‐fat diet (HFD, Beijing HFK Bioscience Co., Ltd, H10141) for 12 or 20 weeks. Mice were kept at 22°C with 45% humidity on a 12‑h light/dark schedule. Given the well‑known inhibitory effect of oestrogens on AS induced by HFD,^30^ the current study exclusively included male mice to enhance plaque development.

### 2.23 Oil Red O staining

We collected aortas from mice on a HFD for 12 or 20 weeks and subjected them to *en face* Oil Red O (ORO) staining. The procedure included: (1) careful removal of fat and adventitial tissue under a dissecting microscope; (2) longitudinal opening of the aorta to expose the intimal surface; (3) staining with freshly prepared ORO; and (4) removal of excess dye using methanol. Plaque areas were analysed using Image Pro Plus software (version 6.0; RRID:SCR_016879, Media Cybernetics, Inc.).

### 2.24 Atherosclerotic lesion assay

We embedded hearts from HFD‐induced mice in paraffin. The aortic root (from the valve leaflet origin to the ascending aorta) was serially sectioned at 5 µm. For lesion quantification, we collected three sections at 80 µm intervals, starting from the first appearance of all three valve leaflets. Paraffin blocks were sectioned at 5‐µm thickness for haematoxylin and eosin (H&E) staining. H&E staining was conducted using an automated stainer. Quantification of plaque area and necrotic core area was performed on H&E stained sections with ImageJ software (RRID:SCR_003070).

Immunofluorescence (IF) staining began with dewaxing and antigen retrieval of paraffin sections. Blocking was performed with goat serum buffer (1 h). Next, primary antibodies were added and incubated overnight at 4°C. The sections were rewarmed to 37°C for 1 h, washed with PBS, and subsequently incubated with fluorophore‑conjugated secondary antibodies at 37°C for 1 h. Finally, DAPI was applied for nuclear visualisation (Solarbio). Negative controls were prepared using species‐matched IgG isotype antibodies at equivalent concentrations in place of the primary antibodies. Fluorescence signals were acquired using a confocal laser scanning microscope (Leica Microsystems) under consistent exposure time and contrast settings across all sections. The primary antibodies were anti‐SMA (Proteintech, 14395‐1‐AP, mouse, 1:400), anti‐CD68 (Proteintech, 28058‐1‐AP, rabbit,1:300) and anti‐TNS1 (Proteintech, 220054‐1‐AP, rabbit 1:300). The quantitative analysis of IF images was performed using Image Pro Plus software version 6.0 (RRID:SCR_016879).

### 2.25 Abstract figure generation instructions

The figure abstract of this article was partially created with BioGDP.com.^31^

### 2.26 Vulnerability plaque index

Vulnerability plaque index (VPI) was calculated according to the formula described in the previous study.^32^ The formula for VPI = (% necrotic core area + % CD68 area)/(% α‐SMA area + % collagen area).

### 2.27 Statistical analysis

scRNA‐seq datasets, bulk RNA‐seq and microarray gene expression data processing, statistical analysis and visualisation were conducted using R 4.4.2 software. To determine whether differences between two groups were statistically significant, we applied a two‑tailed unpaired *t*‑test. All statistical analyses of experimental data were performed using GraphPad Prism (version 9.0). A *p* value below .05 was regarded as statistically significant.

## 3 RESULTS

### 3.1 Macrophage‐like SMC is a key SMC subtype for mouse AS progression and plaque instability

**FIGURE 1:**
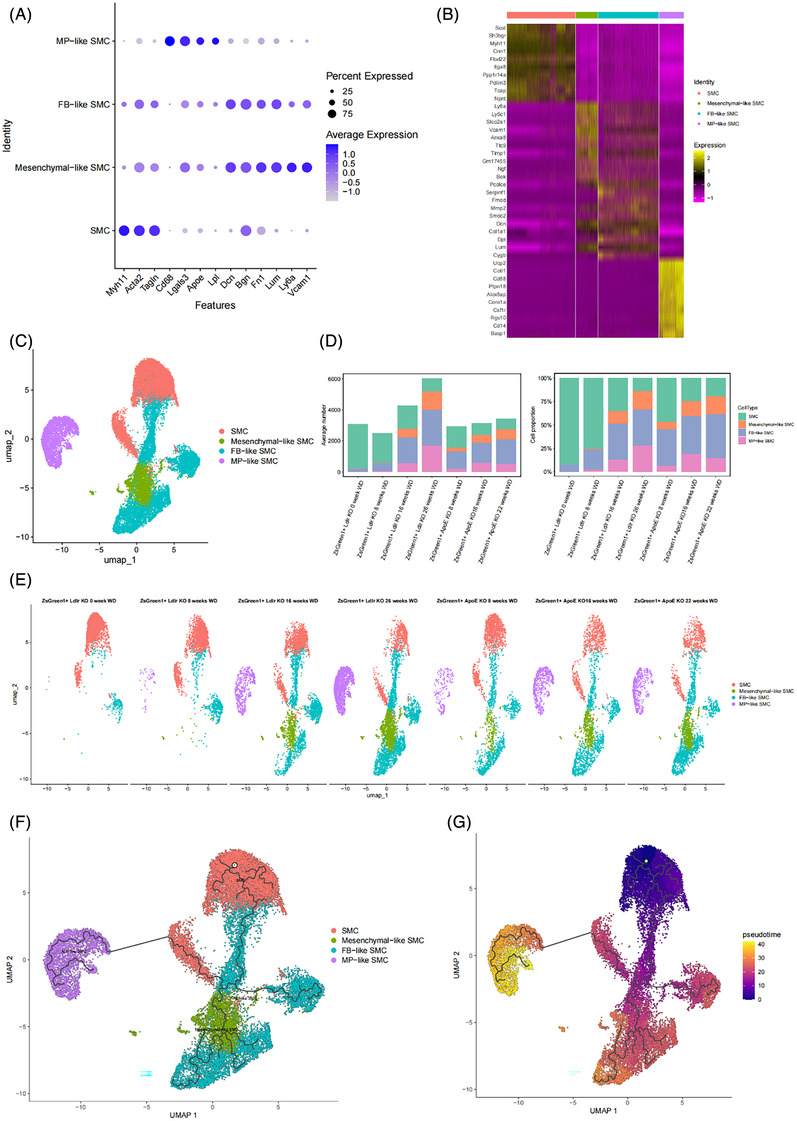
The landscape of smooth muscle cell (SMC) composition of scRNA‐seq dataset of GSE155513 deriving from experimental atherosclerosis model on *Ldlr* ^−/−^ or *ApoE* ^−/−^ background. (A) Dotplot showing the expression of marker genes for each SMC subtype. Marker gene: SMC (*Myh11*, *Act2* and *Tagln*), MP‐like SMC (*Cd68*, *Lgals3*, *Apoe* and *Lpl*), mesenchymal‐like SMC (*Vcam1* and *Ly6a*), fibroblast (FB)‐like SMC (*Fn1*, *Lum*, *Dcn* and *Bgn*). (B) Heatmap showing average expression of top10 genes in each SMC subtype. (C) Uniform manifold approximation and projection (UMAP) of scRNA‐seq data of the mouse AS model exhibiting SMC subtypes. (D) Bar chart showing the difference in average number and cell proportions between mouse AS models with different induction times of high‐fat diet (HFD). (E) UMAP demonstrating the composition of SMCs in mouse models on *Ldlr* ^−/−^ background induced by HFD at 0 week, 8 weeks,16 weeks and 26 weeks or mouse models on *ApoE* ^−/−^ background induced by HFD at 8 weeks, 16 weeks and 22 weeks. (F) Trajectory inference between SMC subtypes predicted by Monocle3. (G) Pseudotime projections between SMC subtypes predicted by Monocle3. (H) Dynamic transcriptomic shifts along the contractile vascular smooth muscle cell (VSMC)‐to‐macrophage (MP)‐like SMC trajectory. The *x*‐axis represents the inferred pseudotime, and the *y*‐axis represents the log‐transformed normalised expression levels. Each dot represents a single cell, coloured by its identified cell type. Solid lines represent the locally weighted scatterplot smoothing (LOESS) fit for each gene. A clear molecular continuum is observed, characterised by the progressive down‐regulation of *Acta2* and *Tagln*.

To identify key SMC subtype contributing to the progression of AS, we reanalysed scRNA‐seq profiles (GSE155513) of lineage‐traced VSMCs from experimental AS at early, mid and late‐stage on *Ldlr* ^−/−^ or *ApoE* ^−/−^ background. scRNA‐seq dataset of GSE15513 were extracted from the samples of SMCs with fluorescent markers for re‐analysis. After quality control and using the CCA integration pipeline to effectively remove technical batch effect, a total of 25 422 SMCs were obtained. Through the standard workflow of the Seurat platform, including dimensionality reduction, clustering and manual annotation, we manually annotated and classified four major SMC clusters based on known marker genes (Figure 1A), namely, contractile SMCs, mesenchymal‐like SMCs, FB‐like SMCs and MP‐like SMCs (Figure 1C). Consistent with previous studies,^14^, ^33^ contractile VSMCs highly expressed marker genes, including *Myh11*, *Tagln* and *Acta2* (Figure 1A), and gradually lost the expression of contractile VSMC marker genes as transdifferentiating into other subtypes during the progression of AS. Mesenchymal‐like SMCs highly expressed marker genes *Ly6a* and *Vcam1*,^15^ FB‐like SMCs highly expressed marker genes, such as *Dcn*, *Bgn*, *Fn1* and *Lum*,^14^ and MP‐like SMCs highly expressed classic MP and foam cell marker genes, such as *Cd68*, *Lgals3,Apoe* and *Lpl* ^14^, ^34^ (Figure 1A). The Findallmarker function was performed to display the top 10 highly expressed genes in each SMC subgroup to confirm the accuracy of clustering (Figure 1B). The number of contractile SMCs in both *ApoE* ^−/−^ and *Ldlr* ^−/−^ mouse models gradually decreased during the progression of AS (Figure 1D,E). FB‐like SMCs began to appear at 8 weeks and became the most numerous subtypes of SMC at both 18 and 26 weeks in *Ldlr* ^−/−^ mouse; In *ApoE* ^−/−^ mouse, the number of FB‐like SMCs reached a considerable level at 8 weeks, and continued to increase at 18 and 22 weeks (Figure 1D,E). A fraction of MP‐like SMCs appeared at 8 weeks and continued to drastically increase, thereafter reaching the maximum at 26 weeks in *Ldlr* ^−/−^ mouse, while the number of MP‐like SMCs was the highest at 16 weeks or at 22 weeks in *ApoE* ^−/−^ mouse (Figure 1D,E). The number of FB‐like SMCs significantly increased only in the progression of *Ldlr* ^−/−^ mouse AS model, while the number of MP‐like SMCs significantly increased in the progression of both *Ldlr* ^−/−^ and *ApoE* ^−/−^ mouse AS models, indicating the potential critical role of MP‐like SMCs in the advanced atherosclerotic plaques.

A potential transdifferentiation trajectory was identified using unbiased trajectory inference and pseudotime analysis, spanning from contractile SMCs at one end to MP‐like SMCs at the other, leaving mesenchymal‐like SMCs and FB‐like SMCs in the middle (Figure 1F). The pseudotime trajectory analysis also confirmed the above‐mentioned VSMC transdifferentiation, starting from the contractile VSMC with the highest expression of contractile marker genes, gradually transdifferentiating, and finally into MP‐like SMCs no longer expressing the classic contractile VSMC marker genes and highly expressing MP marker gene *Cd68* (Figures 1F–H and S7F,G).

To further confirm the accuracy of the SMC subtypes and explore the potential functions of each SMC subtype, GO/KEGG enrichment analyses were carried out on the set of highly expressed genes identified within each SMC subgroup (Figure S1A–E). The contractile VSMCs had related pathways such as muscle contraction (Figure S1A), contractile muscle fibre (Figure S1C) and vascular smooth muscle contraction (Figure S1D). FB‐like SMCs were enriched in extracellular matrix organisation and connective tissue development (Figure S1A), extracellular matrix structural constituent (Figure S1B), extracellular matrix binding (Figure S1B), glycosaminoglycan binding (Figure S1B) and collagen‐containing extracellular matrix (Figure S1C). MP‐like SMCs had related pathways such as antigen processing and presentation (Figure S1A), leukocyte migration (Figure S1A), MHC class II protein complex binding and immunoglobulin receptor binding (Figure S1B), late endosome, lysosomal membrane and endocytic vesicle (Figure S1C) and phagosome (Figure S1D), indicating a classic type of pro‐inflammatory MP‐like SMCs obtaining some of the phagocytic characteristics.

To assess the pathogenic role of each SMC subtype in advanced plaques, we performed ssGSEA to extract SMC sub‐cluster‐specific feature genes from scRNA‐seq dataset GSE155513 as scoring signature and scored them in three datasets of bulk transcriptomic profiles from human vulnerable plaques. The scores defined by MP‐like SMCs increased significantly and consistently in the advanced group (GSE28829), unstable group (GSE120521) and IPH featured group (GSE163154; Figure S2A–C), suggesting that MP‐like SMCs might have the leading pathogenic contributions to atherosclerotic plaque instability. Taken together, these findings revealed that MP‐like SMC was a key SMC subtype for AS progression and plaque instability.

### 3.2 Validation of the effect of MP‐like SMCs on unstable human atherosclerotic plaques

To validate the leading pathogenic contributions of mouse MP‐like SMCs to plaque instability in unstable human atherosclerotic plaques, we reanalysed the scRNA‐seq dataset of GSE253903 from human carotid atherosclerotic plaques (seven symptomatic AS patients and eight asymptomatic AS patients). After the same processing procedure, 10 cell types were identified (Figure S3A,C), including VSMC, endothelial cell (EC), mononuclear MP (Mono/Mac), neutrophil (Neu), FB, mast cell (Mast cell), natural killer cell (NK), T cell and B cell. Figure S3B showed the proportion of each cell type.

**FIGURE 2:**
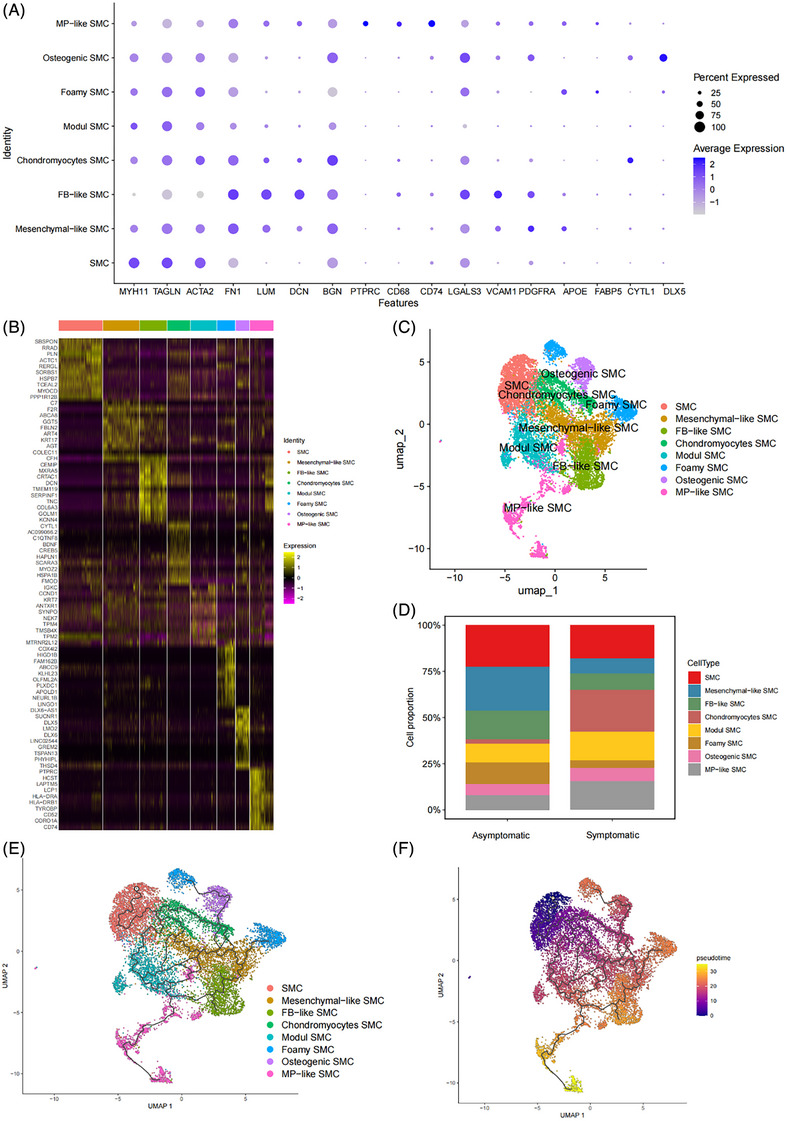
The landscape of smooth muscle cell (SMC) composition of scRNA‐seq dataset of GSE253903 deriving from unstable human atherosclerotic plaques. (A) Dotplot showing the expression of marker genes for each SMC subtype. Marker gene: SMC (*MYH11*, *ACTA2* and *TAGLN*), macrophage (MP)‐like SMC (PTPRC, *CD68* and CD74), mesenchymal‐like SMC (*VCAM1* and *PDGFRA*), fibroblast (FB)‐like SMC (*FN1*, *LUM*, *DCN* and *BGN*), chondromyocyte SMC (C*YTL1*), osteogenic SMC (*DLX5*). (B) Heatmap showing average expression of TOP10 genes in each SMC subtype. (C) Uniform manifold approximation and projection (UMAP) of scRNA‐seq data of human atherosclerotic plaques exhibiting SMC subtypes. (D) Bar chart showing the difference in SMC subtype proportions between symptomatic patients and asymptomatic patients. (E) Trajectory inference between SMC subtypes predicted by Monocle3. (F) Pseudotime projections between SMC subtypes predicted by Monocle3.

We isolated VSMCs and conducted a subdivision of the SMC subtypes. After re‐dimensionality reduction clustering and manual annotation, VSMCs were divided into eight clusters (Figure 2A,C), namely, contractile VSMC (SMC), mesenchymal‐like SMC (*VCAM1* and *PDGFRA*), FB‐like SMC (*FN1*, *LUM*, *DCN* and *BGN*), chondromyocyte SMC (*CYTL1*), osteoblast‐like SMC (*DLX5*), foamy SMC (*APOE* and *FABP5)*, MP‐like SMC (*PTPRC*, *CD68* and *CD74*) and Modul SMC. The Findallmarker function was performed to display the top 10 highly expressed genes in each SMC subgroup to confirm the accuracy of clustering (Figure 2B). There was a significant reduction in the number of contractile SMC, foamy SMC, FB‐like SMC and mesenchymal‐like SMC, while the numbers of chondromyocyte SMCs and MP‐like SMC significantly increased in symptomatic patients (Figure 2D), compared with asymptomatic patients. Pseudotime trajectory analysis of VSMCs demonstrated a progressive contractile SMC transdifferentiation into mesenchymal‐like SMC, followed by divergence into three branches: one branch transdifferentiated into osteoblast‐like SMC and finally into chondromyocyte SMC, the other transdifferentiated into FB‐like SMC, and another branch transdifferentiated into foamy SMC and finally into MP‐like SMC (Figure 2E,F). This observation aligned with mouse findings, where MP‐like SMC consistently occupied the terminal end of the pseudotime trajectory.

GO/KEGG analysis of the highly expressed genes for each SMC subgroup was performed to further confirm the accuracy of the SMC subtypes and explore the potential functions of each SMC subtype. Contractile SMC had processes such as contractile muscle fibre (Figure S4B), vascular smooth muscle contraction and cytoskeleton in muscle cells (Figure S4D). Endodermal cell differentiation and gliogenesis (Figure S4A), platelet‐derived growth factor binding (Figure S4C) and PI‐3K Akt signalling pathway (Figure S4D) in mesenchymal‐like SMC were more active. Consistent with mouse FB‐like SMC, human FB‐like SMC also had processes such as extracellular matrix organisation (Figure S4A), glycosaminoglycan binding and extracellular matrix structural constituent (Figure S4C). Chondromyocyte SMC was enriched in the ossification (Figure S4A) and integrin binding (Figure S4C) and extracellular matrix organisation pathways (Figure S4E). MP‐like SMC was associated with immune cell processes, such as antigen processing and presentation and regulation of T cell activation (Figure S4A), endocytic vesicle and endocytic vesicle membrane (Figure S4B), MHC class II protein complex binding and immune receptor activity (Figure S4C), antigen processing and presentation and phagosome (Figure S4D), and MHC class II antigen presentation and interferon gamma signalling (Figure S4E).

To validate pathogenic contributions of each SMC subtype to unstable plaques, we also performed ssGSEA to extract SMC sub‐cluster‐specific feature genes from scRNA‐seq dataset GSE253903 as scoring signature and scored them in three datasets of bulk transcriptomes gene expression from unstable human atherosclerotic plaques. The scores defined by MP‐like SMCs also increased significantly and consistently in the advanced group (GSE28829), unstable group (GSE120521) and IPH featured group (GSE163154; Figure S5A–C), validating that MP‐like SMC indeed had the leading pathogenic contributions to unstable human atherosclerotic plaques.

### 3.3 HdWGCNA analysis for MP‐like SMC identified the key gene modules involved in unstable atherosclerotic plaques

The re‐analysis of scRNA‐seq datasets of GSE15513 and GSE253903 demonstrated that MP‐like SMC had the leading pathogenic contributions to the progression of AS and unstable atherosclerotic plaques. To identify the key genes of MP‐like SMC that might lead to unstable atherosclerotic plaques, we performed hdWGCNA analysis for MP‐like SMC in the GSE253903 data. When the soft threshold was 9 (Figure 3A), there were a total of 22 modules (Figure 3C). The hub genes of each module were shown in Figure 3C. The green module had the lowest expression in contractile SMC and the highest expression in MP‐like SMC, while the blue module had the highest expression in contractile SMC and the lowest expression in MP‐like SMC (Figure 3D). Figure 3E,F showed the top 25 hub genes of blue module and green module, respectively. Blue module contained known contractile VSMC marker genes (Figure 3E), such as *CNN1*, *ACTA2* and *TAGLN*.^35^ Green module contained known MP marker gene *CD68* and *CD74* ^35^, ^36^ (Figure 3F). We then performed GO/KEGG analysis on the top 100 genes of these two modules. The genes of blue module were enriched in muscle contraction (Figure 3G), focal adhesion (Figure S6A), actin binding (Figure S6C) and cytoskeleton in muscle cells (Figure S6E). The genes in green module were involved in processes such as antigen processing and presentation (Figure 3H), lysosomal membrane (Figure S6B), immune receptor activity (Figure S6D), lysosome, phagosome, inflammatory bowel disease (Figure S6F), indicating that green module were related to the function of MP‐like SMCs.

FIGURE 3High‐dimensional weighted gene co‐expression network analysis (hdWGCNA) analysis identified the key gene modules involved in atherosclerotic unstable plaques. (A) Optimal soft threshold selection. (B) Construction of co‐expression network using the optimal soft threshold of 10, with genes being divided into 22 modules and resulting in a dendrogram. The upper part was the hierarchical clustering tree of genes, and the lower part was gene module, namely, network module. (C) Eigengene‐based connectivity (kME) for each gene was calculated in co‐expression network analysis to identify highly connected genes (hub genes) within each module. (D) Module activity in smooth muscle cell (SMC) subtypes. (E) Blue module network plot showing the kME top 25 gene. Each node represents a gene, and each edge represents the co‐expression relationship between two genes in the network. (F) Green module network plot showing the kME top 25 gene. Each node represents a gene, and each edge represents the co‐expression relationship between two genes in the network. (G) Gene Ontology (GO) analysis (BP) results of top100 genes in blue module. (H) GO analysis (BP) results of top100 genes in green module. (I) The violin plot showing the expression levels of the blue module in SMC and MP‐like SMC of symptomatic patients and asymptomatic patients. The harmonised module eigengene (hME) on the *Y*‐axis represents the expression level of this module. (J) The violin plot showing the expression levels of the green module in SMC and MP‐like SMC of symptomatic patients and asymptomatic patients. The hME on the *Y*‐axis represents the expression level of this module.

**Figure:**
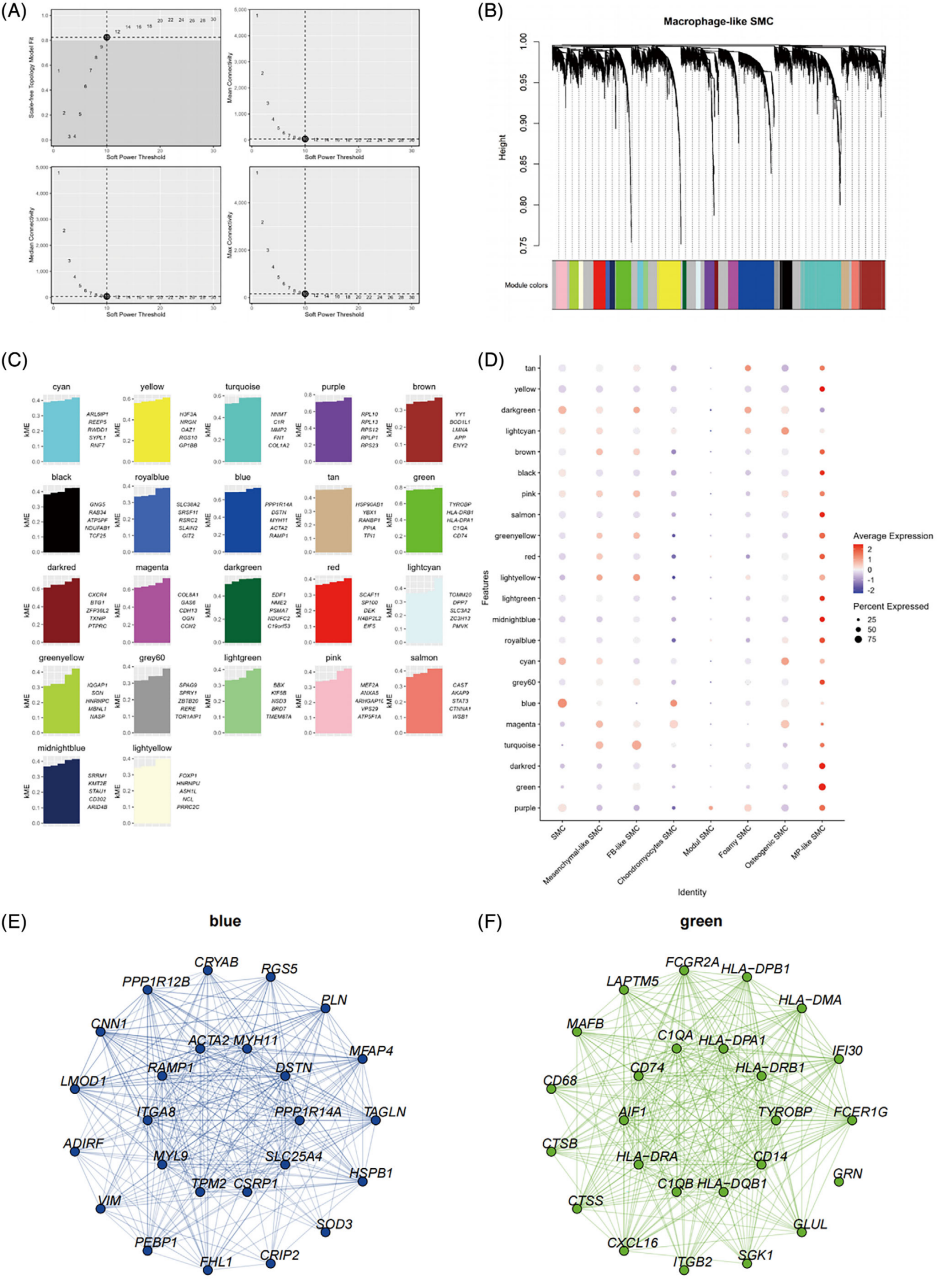

**Figure:**
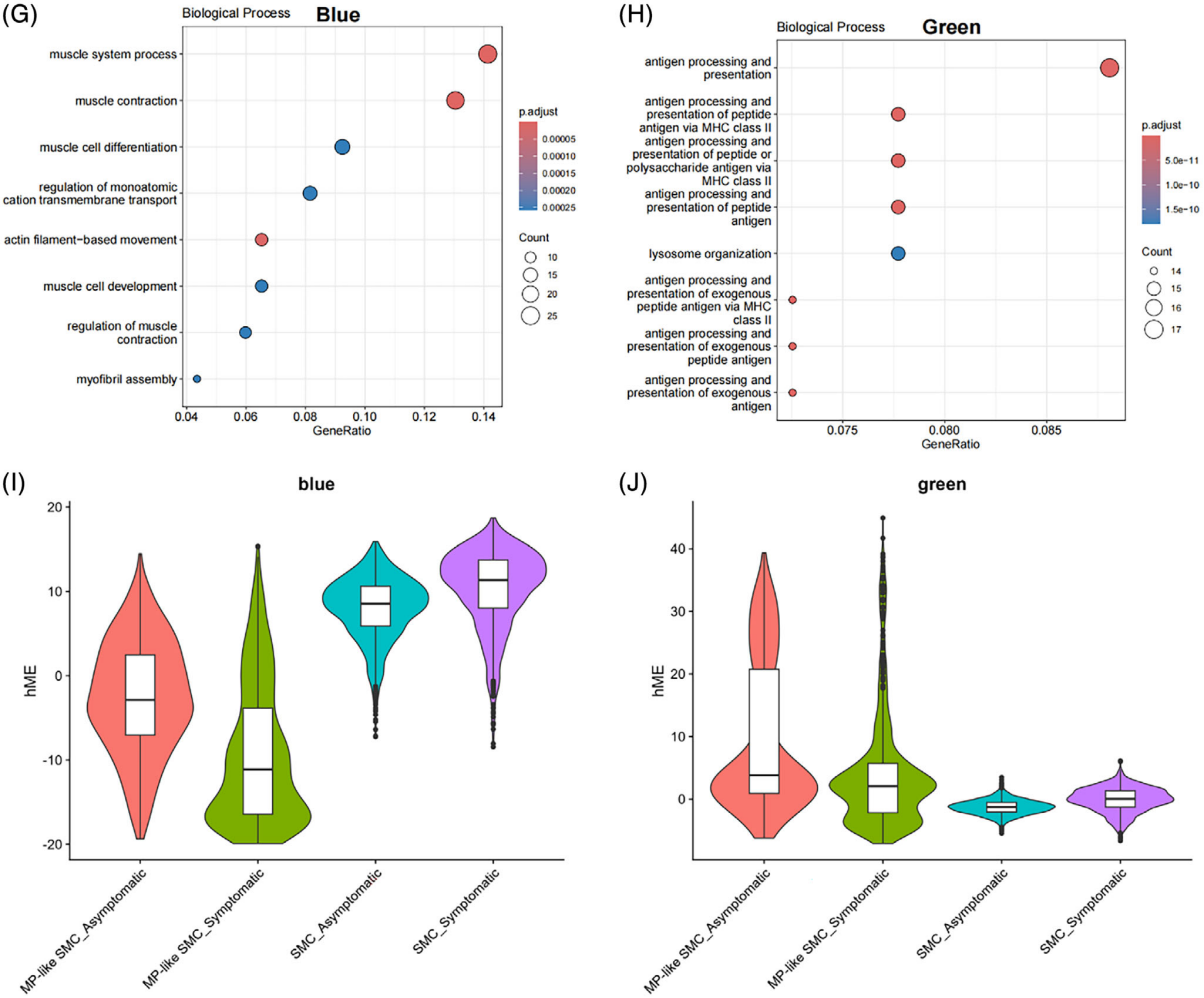

Next, we examined the expression of these two modules in contractile VSMC and MP‐like SMC of symptomatic and asymptomatic patients, respectively. The expression of blue module genes in MP‐like SMC was lower in symptomatic patients than in asymptomatic patients (Figure 3I), while there was no significant difference in the expression of green module genes in MP‐like SMC between symptomatic patients and asymptomatic patients (Figure 3J), indicating that blue module genes might play an important role in contractile VSMC transdifferentiation into MP‐like SMC and unstable atherosclerotic plaques. Therefore, the blue module was selected for subsequent analysis.

According to the above analysis, we conducted differential gene analysis of MP‐like SMCs in scRNA‐seq dataset GSE253903 (*p* < .05, log2FC ± >.5). The gene list (Table S3) obtained from screening was crossed with blue module, and a total of 26 genes were obtained (Table S4), the expression of which was all decreased in symptomatic patients. Among the 26 genes, apart from contractile VSMC marker genes (*ACTA2*, *MYH11*, *CNN1*, *TAGLN* and *MYL9*), there were also some previously reported genes, such as *CSRP1*, which had been confirmed to have a lower expression in the state of more severe AS lesions. Interestingly, the expression of *CSRP1* in stable lesions seemed to be enriched in fibrous caps, while its expression disappeared in unstable or ruptured cap structure.^37^ ITGA8(*α8)* had been proven to be down‐regulated in advanced atherosclerotic lesions. In the ApoE‑deficient background, both α8 heterozygous (α8^+/–^) and α8 knockout (α8^–/–^) mice exhibited significantly more atherosclerotic lesions compared with their α8 wild‑type littermates.^38^ Previous studies have demonstrated that *Rgs5* regulates signalling pathways involved in VSMC differentiation, migration, contraction, as well as tissue inflammation and fibrosis.^39^ Recently, *Rgs5* expression levels were found to be significantly reduced in TNF‑α‑treated VSMCs and in mouse neointimal hyperplasia, whereas high *Rgs5* expression was identified as a homeostasis‑relevant transcriptional signature of VSMCs in healthy arteries, as reported in one study.^40^ Furthermore, the down‐regulation of PLN and LMOD1 genes had been reported to be involved in VSMC phenotype transdifferentiation.^41^ TGFB1I1 was an SRF/myocardin‐regulated contractile VSMC marker,^42^ which is important for keeping smooth muscle cells in a contractile state by preventing their proliferation.

We excluded the previously reported genes mentioned above and screen the remaining 15 genes using LASSO in the dataset of GSE120521 from unstable human atherosclerotic plaques. Eventually, we obtained three genes, *TNS1*, *PPP1R14A* and *COPRS* (Figure S7A,B). We then analysed their expression levels in scRNA‐seq dataset of GSE155513. The expression levels of *Ppp1r14a* and *Tns1* genes changed the most significantly and decreased as the induction time increased, while the expression levels of *Coprs* gene were too low in scRNA‐seq dataset of GSE155513 (Figure S7C–E). It was reported that *Ppp1r14a* gene maintained the contractile phenotype of VSMCs,^43^ therefore, *Tns1* gene was selected for further analysis. Next, we performed pseudotime trajectory analysis on *Tns1* gene, and the expression of Tns1 gene continuously decreased with increasing SMC transdifferentiation into MP‐like SMC marked by continuously increased *Cd68* gene expression, contrary to the expression pattern of contractile VSMC marker genes (Figures 1F–H and S7F,G). The results identified *TNS1* gene as the key gene for MP‐like SMC transdifferentiation and unstable atherosclerotic plaques.

### 3.4 The decreased *TNS1* gene drives contractile VSMC transdifferentiation into MP‐like SMC and regulates intercellular communication

Based on the results of analysis mentioned above, we divided VSMCs into *TNS1*‐positive (*TNS1*‐pos) group and *TNS1*‐negative (*TNS1*‐neg) group to explore the effects of *TNS1* gene expression on the transdifferentiation of contractile VSMC into MP‐like SMC, according to the expression levels of *TNS1* gene in VSMCs in scRNA‐seq dataset GSE253903 (Figure 4A). Compared with the *TNS1*‐pos group, the expression levels of contractile VSMC marker genes (*CNN1*, *MYH11* and *ACTA2*) were significantly decreased (Figure 4B–D), while the expression levels of MP marker gene *CD68* and inflammatory‐related genes, such as *CXCL12*, *CCL2* and *VCAM‐1*, were significantly increased in the *TNS1*‐neg group (Figures 4E and S8C–E). Accordingly, the expression levels of *KLF4* gene, encoding key TF promoting VSMC phenotype switching,^44^, ^45^, ^46^ were significantly increased in the *TNS1*‐neg group (Figure 4F), while the expression levels of *SRF* and *MYCOD* genes, encoding key TFs maintaining the contractile phenotype of VSMCs,^47^, ^48^, ^49^, ^50^ were significantly decreased (Figure 4G,H). These results demonstrated that the decreased expression of *TNS1* gene not only initiated VSMC phenotype switching, but also drived contractile VSMC transdifferentiation into MP‐like SMC probably via down‐regulating *SRF* and *MYOCD* genes and up‐regulating *KLF4* gene in VSMCs.

FIGURE 4
*TNS1* regulates cell communication networks ligand–receptor pairs associated with vascular smooth muscle cell (VSMC) phenotype switching. (A) Raincloud plots showing the expression levels of *TNS1* (VSMC grouping basis of *TNS1*, defined the *TNS1‐*positive group using the top quartile (75th percentile) of expression to ensure that our ‘positive’ population consists of cells with robust transcriptional activity of the gene). The half‐violin plots represent the probability density of gene expression, highlighting the multi‐modal distribution across cells. Each dot represents an individual cell, with jittering applied to minimise overlap. Central boxplots indicate the median, interquartile range (IQR) and 95% confidence intervals of the expression distribution. log2FC values are indicated in the subtitles. *p* values were calculated using the Wilcoxon rank‐sum test. (B–H) Raincloud plots showing the expression levels of contractile VSMC marker genes (*CNN1*, *MYH11* and *ACTA2*), macrophage marker gene *CD68* and key transcription factor genes (*KLF4*, *SRF* and *MYOCD*) in *TNS1*‐pos and *TNS1*‐neg groups. The half‐violin plots represent the probability density of gene expression, highlighting the multi‐modal distribution across cells. Each dot represents an individual cell, with jittering applied to minimise overlap. Central boxplots indicate the median, interquartile range (IQR) and 95% confidence intervals of the expression distribution. log2FC values are indicated in the subtitles. *p* values were calculated using the Wilcoxon rank‐sum test. (I–L) Density scatter plots showing the correlation between *TNS1* expression and VSMC phenotype related pathway activation, including smooth muscle contraction (I), response to oxidative stress (J), extracellular matrix disassembly (K) and positive regulation of cholesterol esterification (L). (M‐N) Violin plots demonstrating the distribution of (M) contractile and (N) inflammatory signature scores in *TNS1*‐pos and *TNS1*‐neg VSMC subpopulations. Each score represents the relative enrichment of a multi‐gene module after background subtraction. Mean ΔScore indicates the arithmetic difference in average scores between the two groups. Statistical significance was determined by the Wilcoxon rank‐sum test. (O) The total number of interactions and the interaction intensity of the inferred cell–cell communication networks in *TNS1*‐pos and *TNS1*‐neg groups in VSMCs. (P) Differential heatmaps illustrating the relative changes in the number of interactions (left panel) and interaction strength (right panel) between the two VSMC phenotype states. The differential signalling was calculated as the interaction value in the *TNS1*‐neg group minus that in *TNS1*‐pos cells. Therefore, the orange colour indicates signalling pathways that are enhanced in the *TNS1*‐neg group, whereas the green colour indicates signalling pathways that are enhanced in the *TNS1*‐pos group. The top colour bar plots represent the sum of incoming signalling changes for each cell population acting as receivers, while the right bar plots represent the sum of outgoing signalling changes for cell populations acting as senders. (Q) Comparison of several signalling pathways in *TNS1*‐pos and *TNS1*‐neg groups. (R‐T) Chord plots showing the signalling pathways (PDGF, THBS and CXCL) of aggregated cell–cell communication networks at the signalling pathway level in *TNS1*‐pos and *TNS1*‐neg groups. (U) Chord plots showing the VCAM signalling pathways of aggregated cell–cell communication networks at the signalling pathway level in *TNS1*‐neg group. (V) Chord diagrams showing the ligand–receptor pairs of VCAM signalling pathways in *TNS1*‐neg group. (W) Chord diagrams showing the ligand–receptor pairs of PDGF signalling pathways in *TNS1*‐pos and *TNS1*‐neg groups. (X) Chord diagrams showing the ligand–receptor pairs of CXCL signalling pathways in *TNS1*‐pos and *TNS1*‐neg groups.

**Figure:**
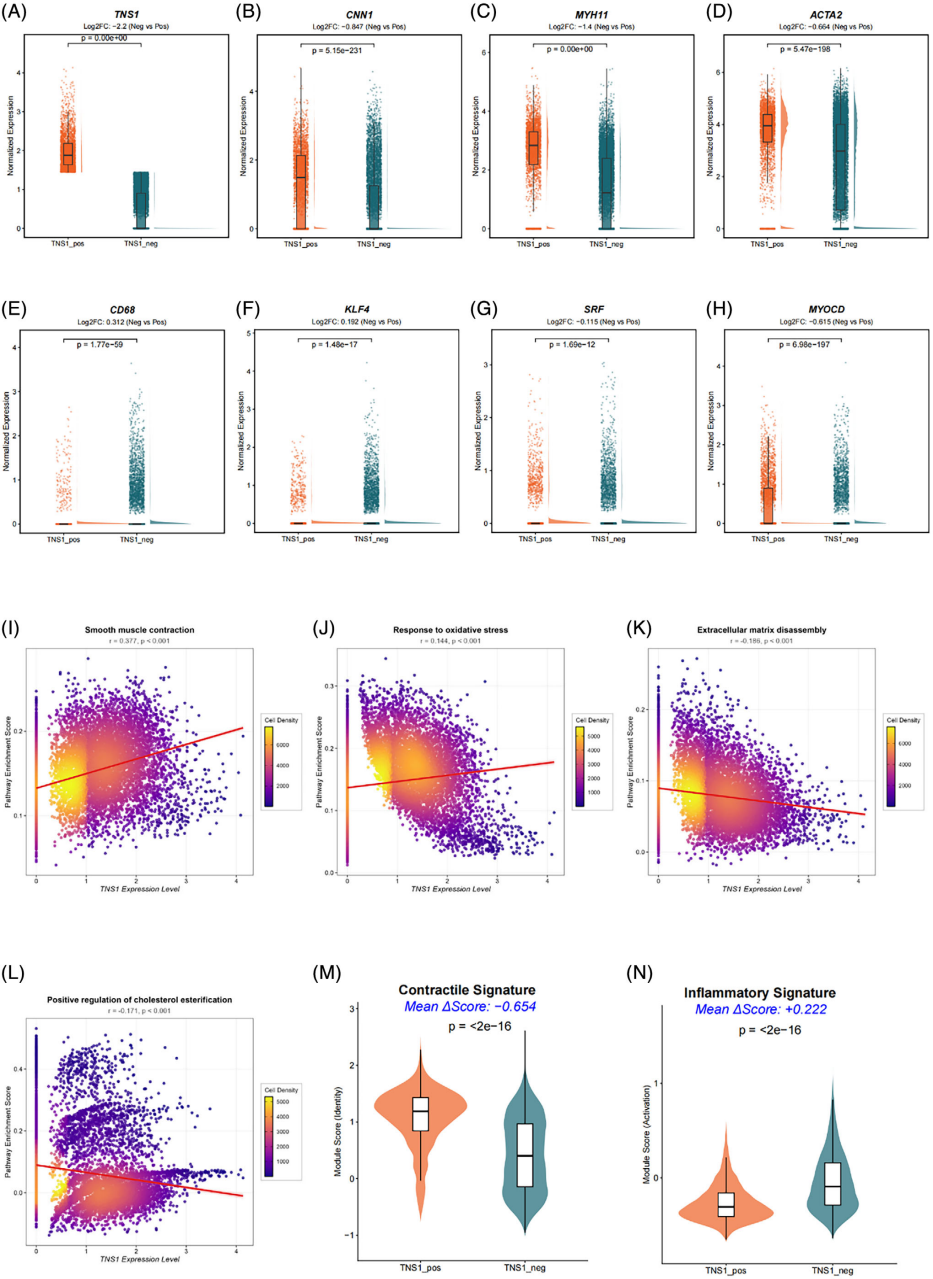

**Figure:**
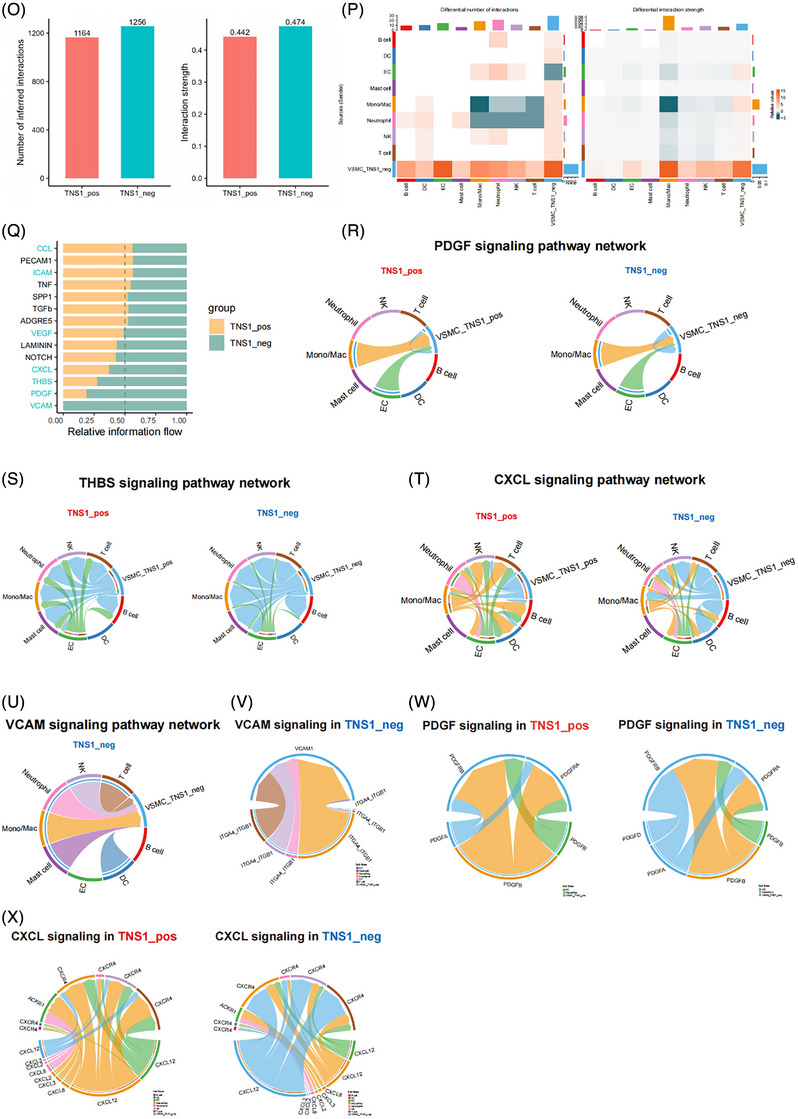

We examined the relationship between the expression levels of *TNS1* gene in VSMCs and signatures of pathways involved in smooth muscle contraction, response to oxidative stress, extracellular matrix disassembly and cholesterol esterification. The expression of *TNS1* gene was positively correlated with smooth muscle contraction (Figure 4I) and the response to oxidative stress (Figure 4J), indicating that the low expression levels of *TNS1* gene might reduce the contraction state of SMCs and the normal processing ability for oxidative stress response to maintain cell homeostasis, thus inducing the inflammatory levels. Furthermore, the expression of *TNS1* gene was negatively correlated with the pathways involved in extracellular matrix disassembly (Figure 4K) and cholesterol esterification (Figure 4L), indicating that the decreased expression levels of *TNS1* gene might lead to intensified extracellular matrix disassembly and enhanced cholesterol esterification enabling the storage of free cholesterol in lipid droplets and contributing to MP‐like SMC transdifferentiation.^51^, ^52^ We further performed a multi‐gene Module Score analysis using the Seurat AddModuleScore framework to quantitatively evaluate the functional effects of the decreased expression levels of *TNS1* gene on the scores of contractile gene signature or inflammatory gene signature in VSMCs. Compared with the *TNS1*‐pos group, the *TNS1*‐neg group showed a dramatic reduction in the contractile score (Mean ΔScore = −.654, *p* < 2e−16, Figure 4M) and a significant elevation in the inflammatory score (Mean ΔScore = +.22, *p* < 2e−16, Figure 4N), indicating that the *TNS1*‐neg group represented a biologically distinct, de‐differentiated VSMC state.

VSMC phenotype switching initiated by the decreased *TNS1* gene and subsequent MP‐like SMC transdifferentiation might lead to alterations of cellular communication between VSMCs or between VSMCs and other types of cells. We compared networks between the *TNS1*‐pos and *TNS1*‐neg groups of VSMCs to explore the effects of *TNS1* gene expression levels on cellular communication in VSMCs. Both the strength and counts of overall cell communication in the *TNS1*‐neg group had increased, compared with the *TNS1*‐pos group (Figure 4O,P), indicating that the down‐regulation of *TNS1* gene in VSMCs might enhance the intercellular communication between VSMCs themselves and other types of cells in the pathogenesis of AS. We then selected the signalling pathways closely related to VSMC phenotype switching and transdifferentiation into other subtypes (Figures 4Q and S8A), including the core growth factor pathways (TGF‐β, PDGF and VEGF), the pathways related to inflammation (TNF, CCL, ICAM, VCAM and CXCL), the pathways related to extracellular matrix and adhesion (SPP1, LAMININ, THBS and PECAM1) and important intercellular communication‐related signalling pathways (including NOTCH and ADGRE5). VCAM, PDGF, THBS and CXCL signalling pathways were more active in the *TNS1*‐neg group than in *TNS1*‐pos group (Figure 4Q). VCAM‐1 was a cell adhesion molecule involved in inflammation and played a significant role in driving the inflammatory response of AS.^53^ The VCAM signalling pathway was present in the *TNS1*‐neg group (Figure 4Q,U,V), but not in the *TNS1*‐pos group (Figure 4Q). The levels of PDGF, THBS and CXCL signalling pathways were higher in the *TNS1*‐neg group than in the *TNS1*‐pos group (Figure 4Q). The PDGF signal pathway, promoting VSMC transdifferentiation into other phenotypes and up‐regulating the levels of inflammatory factors and adhesion molecules,^54^, ^55^ was enhanced in the *TNS1*‐neg group and exerted a reinforcing effect on the signals sent by VSMCs themselves (Figure 4R). Moreover, the THBS and CXCL signal pathways strengthened the intercellular communication between VSMCs and other types of cells in the *TNS1*‐neg group (Figure 4S,T). We further analysed the specific molecular interactions within the PDGF, CXCL and THBS signalling pathways. Different from PDGFA and PGDFB signal, PDGFD signal emerged in the *TNS1*‐neg group, but not in the *TNS1*‐pos group (Figure 4W). PDGFD was strongly expressed in fatty streaks, and increased expression of PDGFD promoted VSMC phenotype switching by inhibiting the expression of multiple contractive VSMC marker genes, including *ACAT2* and *MYH11*.^54^ PDGFD also played an important role in the pathogenesis of AS by stimulating the activity of matrix metalloproteinase‐9 (MMP‐9) and influencing monocyte migration.^55^ CXCL12 enhanced the intercellular communication between mononuclear MPs, T cells and NK cells through CXCR4 in the *TNS1*‐neg group (Figure 4X). It was reported that CXCL12 (SDF‐1) regulated various cellular activities by binding to CXCR4 or CXCR7.^56^ A recent investigation found that CXCL12 levels rose significantly in an in vitro co‑culture system combining THP‑1 and VSMCs, as well as in an in vivo AS model, and CXCL12 released by THP‐1 cells stimulated VSMC proliferation, leading to the progression of AS plaques.^57^ Thus, the down‐regulation of *TNS1* gene in VSMCs might also be associated with increased expression of *CXCL12* gene, thereby promoting AS lesions. THBS‐2 was reported to regulate matrix metalloproteinase‐2 (MMP‐2),^58^ which was associated with the vulnerability of atherosclerotic plaques, thus probably influencing AS progression. The interaction of THBS‐2 signalling pathway between VSMCs and other types of cells was enhanced in the *TNS1*‐neg group (Figure S8B), suggesting that the decreased expression of *TNS1* gene in VSMCs might promote the progression of AS by regulating extracellular matrix degradation through THBS2 signalling pathway. In addition, the expression levels of *PDGFD* gene, *THBS2* gene and its downstream target gene *MMP2* were also increased in the *TNS1*‐neg group, compared with the *TNS1*‐pos group (Figure S8F–H).

In summary, the decreased expression of *TNS1* gene in VSMCs might play a vital role in driving contractile VSMC transdifferentiation into MP‐like SMC and regulate intercellular communication along with AS progression and the final rupture of unstable atherosclerotic plaques.

### 3.5 ZEB1 was identified as transcription factor positively regulating *TNS1* gene in VSMC phenotype switching and transdifferentiation during AS progression

We tried to decipher the underlying mechanism of the down‐regulated *TNS1* gene within VSMCs in human atherosclerotic plaques. The positive feedback loop between the TF ZEB1 and *TNS1* gene in a TGFβ‐induced epithelial–mesenchymal transition cell model^59^ suggested that ZEB1 might also transcriptionally activate *TNS1* gene in VSMCs. We first analysed the expression levels of *TNS1* and *ZEB1* mRNAs in VSMCs of GSE253903. The expression levels of *TNS1* and *ZEB1* mRNAs were lower in VSMCs from symptomatic patients than those from asymptomatic patients (Figure S9A,B), respectively. Compared with the *TNS1*‐pos group, the mRNA levels of *ZEB1* gene were significantly decreased in the *TNS1*‐neg group (Figure S9C). Then we analysed the expression pattern of *Zeb1* gene and performed pseudotime trajectory analysis on *Zeb1* gene in scRNA‐seq datasets of GSE155513. Similar to *Tns1* gene, the expression levels of *Zeb1* gene changed significantly and decreased as the induction time increased (Figure S9D), and the expression of *Zeb1* gene continuously decreased with increasing SMC transdifferentiation into MP‐like SMC in scRNA‐seq datasets of GSE155513 (Figures 1F–H, S7F,G and S9E). The expression levels of *ZEB1* mRNA were also significantly decreased in in vitro cholesterol‐induced model of VSMC transdifferentiation into MP‐like SMC (Figure S9F). These results collectively indicated that the decreased ZEB1 might be responsible for the down‐regulation of *TNS1* gene driving contractile VSMC transdifferentiation into MP‐like SMC during AS progression.

**FIGURE 5:**
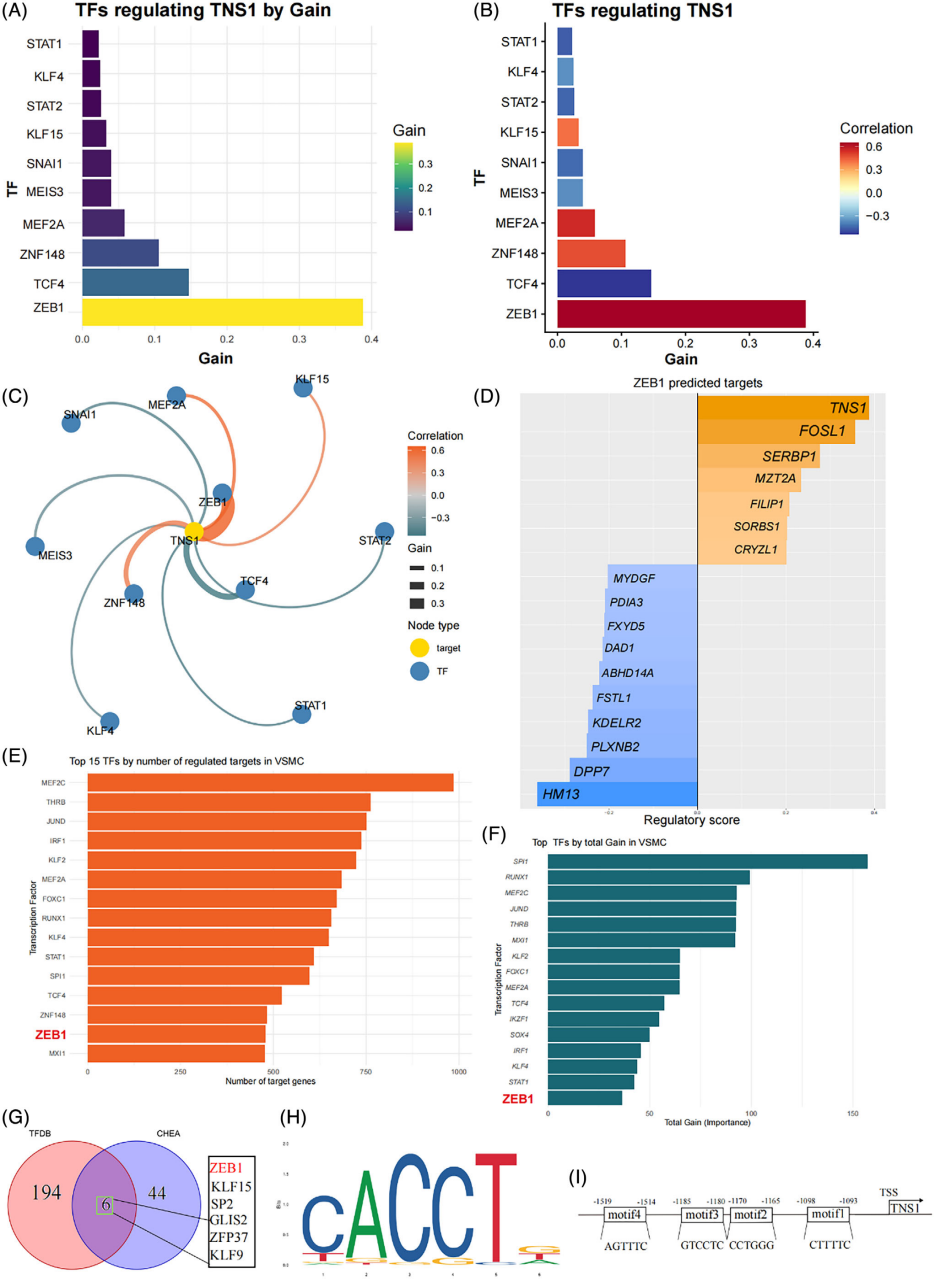
The regulatory relationship between transcription factor ZEB1 and *TNS1* gene. (A) Transcription factors predicted to regulate *TNS1* gene in vascular smooth muscle cells (VSMCs), ranked by total regulatory importance (Gain), a metric from XGBoost models indicating the overall contribution of each transcription factor (TF) to predicting the expression of its target genes. TFs are ordered by increasing Gain (top to bottom). Colour intensity reflects the Gain magnitude, using the Viridis colour scale (from purple for low Gain to yellow for high Gain). (B) TFs regulating *TNS1* gene in VSMCs, coloured by the direction of correlation. This plot presents the same set of TFs as in (A), but with bars coloured according to the Pearson correlation coefficient (Cor) between the TFs and *TNS1* gene expression. Bar length corresponds to the Gain value. TFs are sorted by ascending Gain. This visualisation highlights both the regulatory strength (Gain) and the potential activating (red) or repressive (blue) relationship with *TNS1* gene. (C) The directed network showing *TNS1* gene as the central target node (gold) surrounded by its top 10 predicted TFs (blue). Edges point from each TF to *TNS1* gene and are coloured by the correlation coefficient (blue to red), with edge width proportional to the Gain value (thicker edges indicate stronger importance). The layout is a force‐directed algorithm (‘fr’) to optimally space nodes. (D) The bar plot showing the top predicted target genes of TF ZEB1, as defined by the regulon analysis in high‐dimensional weighted gene co‐expression network analysis (hdWGCNA) in VSMCs. The *x*‐axis represents the regulatory importance score (Gain) derived from XGBoost models, quantifying the contribution of ZEB1 to predict the expression of each target gene. Only target genes with a Gain > .2 are shown. Genes are sorted by descending Gain and are coloured according to the Pearson correlation coefficient (Cor) between ZEB1 and the target gene: orange bars indicate positive correlation (potential activation), blue bars indicate negative correlation (potential repression). The analysis is based on the filtered regulon list obtained using strategy A (top 10 TFs per target gene, reg_thresh = .01) from the VSMC‐specific TF network. This plot reveals the confident downstream targets of ZEB1 in VSMCs. (E) Top TFs ranked by the number of predicted target genes in VSMCs. The *x*‐axis indicates the count of unique target genes assigned to each TF. TFs are sorted in descending order of target count (from top to bottom). The uniform bar colour emphasises the ranking. Data are derived from the hdWGCNA analysis of VSMCs. (F) Bar plot displaying the top TFs with the highest cumulative Gain scores, as derived from the TF regulatory network analysis using hdWGCNA. The *x*‐axis represents the total Gain. (G) Intersection diagram of TFs for *TNS1* gene predicted by TFDB and CHEA databases. (H) Predicted binding site of ZEB1. (I) Four potential binding sites of ZEB1 in the promoter region of *TNS1* gene predicted by the JASPAR database.

To comprehensively investigate the potential TFs of *TNS1* gene in the context of VSMC phenotype switching and transdifferentiation, we conducted an hdWGCNA‐based TF regulatory network analysis in scRNA‐seq dataset of GSE253903 across all SMCs and evaluated the regulatory importance (Gain) of each TF using XGBoost models. The result revealed that ZEB1 exhibited the highest Gain score among all predicted TFs (Figure 5A). Correlation analysis further demonstrated that *ZEB1* gene expression was most strongly and positively correlated with *TNS1* gene expression (Figure 5B). To visualise this regulatory relationship, we constructed a *TNS1*‐centred transcriptional network. Figure 5C clearly showed ZEB1 as the node with the strongest connection (edge width, representing Gain value) to *TNS1* gene. These results suggested that ZEB1 might act as a core transcriptional activator of *TNS1* gene. Conversely, we examined the regulon of ZEB1 itself. Using a stringent cutoff (Gain > .2), the ZEB1 regulon included *TNS1* gene as one of its top target genes, again with a positive correlation (Figure 5D). This reciprocal finding further supported a ZEB1‐*TNS1* regulatory axis in VSMCs. We next asked whether ZEB1 might function as a global regulator in VSMCs. We ranked all TFs by two complementary metrics: the number of unique target genes assigned to each TF (Figure 5E) and the total cumulative Gain (Figure 5F). In both analyses, ZEB1 ranked among the top TFs, comparable to KLF4, a well‑established master regulator promoting VSMC phenotype switching. We further identified six candidate TFs using Animal TFDB and CHEA datasets in the promoter of *TNS1* gene, including ZEB1 (Figure 5G), verifying the regulatory relationship between ZEB1 and *TNS1* gene. We used JASPAR to predict the binding region of ZEB1 to the promoter of *TNS1* gene, and the results revealed four potential TF binding sites (Figure 5H,I). Collectively, ZEB1 was identified as a key TF transcriptionally regulating *TNS1* gene in VSMC phenotype switching and transdifferentiation during AS progression.

### 3.6 The decreased *TNS1* gene expression in human plaques

To begin addressing the role of *TNS1* gene in the pathogenesis of AS, we initially assessed *TNS1* mRNA levels using publicly available datasets of arterial tissues from the GTEx project. *TNS1* mRNA is highly expressed in human aortic and coronary tissues (Figure S10A), indicating a potential role of *TNS1* gene in arterial function. Compared with healthy arteries, *TNS1* mRNA was significantly reduced in human atherosclerotic arteries (Figure 6A,B), indicating the involvement of *TNS1* gene in the pathogenesis of AS. Most importantly, *TNS1* mRNA was even lower in advanced plaques than in early plaques (Figure 6C–E), or in unstable plaques than in stable plaques (Figure 6F,G), suggesting that the decreased *TNS1* gene expression might have a promotive role in the unstable plaques. Consistent with mRNA findings, the expression levels of TNS1 protein were also significantly decreased in human plaques, compared with the internal mammary artery (IMA; Figure 6H,I), and furthermore, the TNS1 protein signals co‐localising with α‐SMA^+^ VSMCs were lower in human plaques than those in IMA (Figure 6H,J). The expression levels of TNS1 protein were significantly decreased in advanced plaques, compared with early plaques (Figure 6K,L), and furthermore, the TNS1 protein signals co‐localising with α‐SMA^+^ VSMCs were lower in advanced plaques than those in early plaques (Figure 6K,M). These observations suggested that down‐regulation of *TNS1* gene in VSMCs in human atherosclerotic plaques would have important roles in AS progression and the final rupture of unstable atherosclerotic plaques. Re‐analysis of GSE181362 and GSE47744 revealed the markedly decreased expression of *TNS1* gene in in vitro cholesterol‐induced model of MP‐like SMC transdifferentiation (Figure S10B,C). TNS1 knockdown resulted in significantly elevated levels of MP marker LGALS3 in HASMCs (Figure S11), indicating the involvement of the decreased *TNS1* gene expression in VSMC transdifferentiation into MP‐like SMC.

FIGURE 6The expression pattern of *TNS1* gene in atherosclerotic plaques. (A) Re‐analysis of *TNS1* gene expression in microarray datasets (GSE100927; control tissue = 35, AS = 69). Each dot represents an individual sample. Data were expressed as medians and interquartile ranges. (B) *TNS1* gene expression levels in atherosclerotic (*n* = 436) and normal (*n* = 538) arterial tissues of human samples deposited at the GTEx Portal (gtexportal.org). Each dot represents an individual sample. Data were expressed as medians and interquartile ranges. Statistical comparisons were performed using the Wilcoxon rank‐sum test to account for the non‐normal distribution of TPM values. (C) *TNS1* expression across human artery in the GTEx database (healthy = 523, mild = 164, severe = 42). Each dot represents an individual sample. Data were expressed as medians and interquartile ranges. Statistical comparisons were performed using the Wilcoxon rank‐sum test to account for the non‐normal distribution of TPM values. (D) Re‐analysis of *TNS1* gene expression in microarray datasets (GSE43292; stages I and II = 32, stage IV and over = 32). Each dot represents an individual sample. Data were expressed as medians and interquartile ranges. (E) Re‐analysis of *TNS1* expression in microarray datasets (GSE28829; early carotid AS plaque = 13, advanced carotid AS plaque = 16). Each dot represents an individual sample. Data were expressed as medians and interquartile ranges. (F) Re‐analysis of *TNS1* expression in RNA‐seq datasets (GSE120521; stable carotid AS plaque = 4, unstable carotid AS plaque = 4). Each dot represents an individual sample. Data were expressed as medians and interquartile ranges. (G) Re‐analysis of *TNS1* expression in microarray datasets (GSE163154; non‐IPH carotid AS plaques = 16, IPH carotid AS plaques = 27). Each dot represents an individual sample. Data were expressed as medians and interquartile ranges. (H) Representative images of TNS1 protein in internal mammary artery (IMA) and carotid plaques, detected by double immunofluorescence staining with antibodies against TNS1 and the vascular smooth muscle cell (VSMC) marker α‐SMA, respectively. Cell nuclei were stained with DAPI (Blue). Scale bars, 100 µm. Original magnification, ×400. (I) The statistical result of fold difference in TNS1 protein levels in IMA and carotid plaques in H. *n* = 6 for each group. (J) The statistical result of fold difference in TNS1 protein levels in VSMCs in IMA and carotid plaques in H. *n* = 6 for each group. (K) Representative images of TNS1 protein in human carotid artery early plaque and advanced plaque detected by double immunofluorescence staining with antibodies against TNS1 and the VSMC marker α‐SMA, respectively. Cell nuclei were stained with DAPI (Blue). Scale bars, 100 µm. Original magnification, ×20. (L) The statistical result of fold difference in TNS1 protein levels in human carotid artery early plaque and advanced plaque in K. *n* = 5 for early plaque group, and *n* = 8 for advanced plaque group. (M) The statistical result of fold difference in TNS1 protein levels in VSMCs in in human carotid artery early plaque and advanced plaque in K. *n* = 5 for early plaque group, and *n* = 8 for advanced plaque group. Data in I, J, L and M were expressed as mean ± SEM. Results in A, D, E, F, G, I, J, L and M were evaluated by two‐tailed unpaired *t*‐test.

**Figure:**
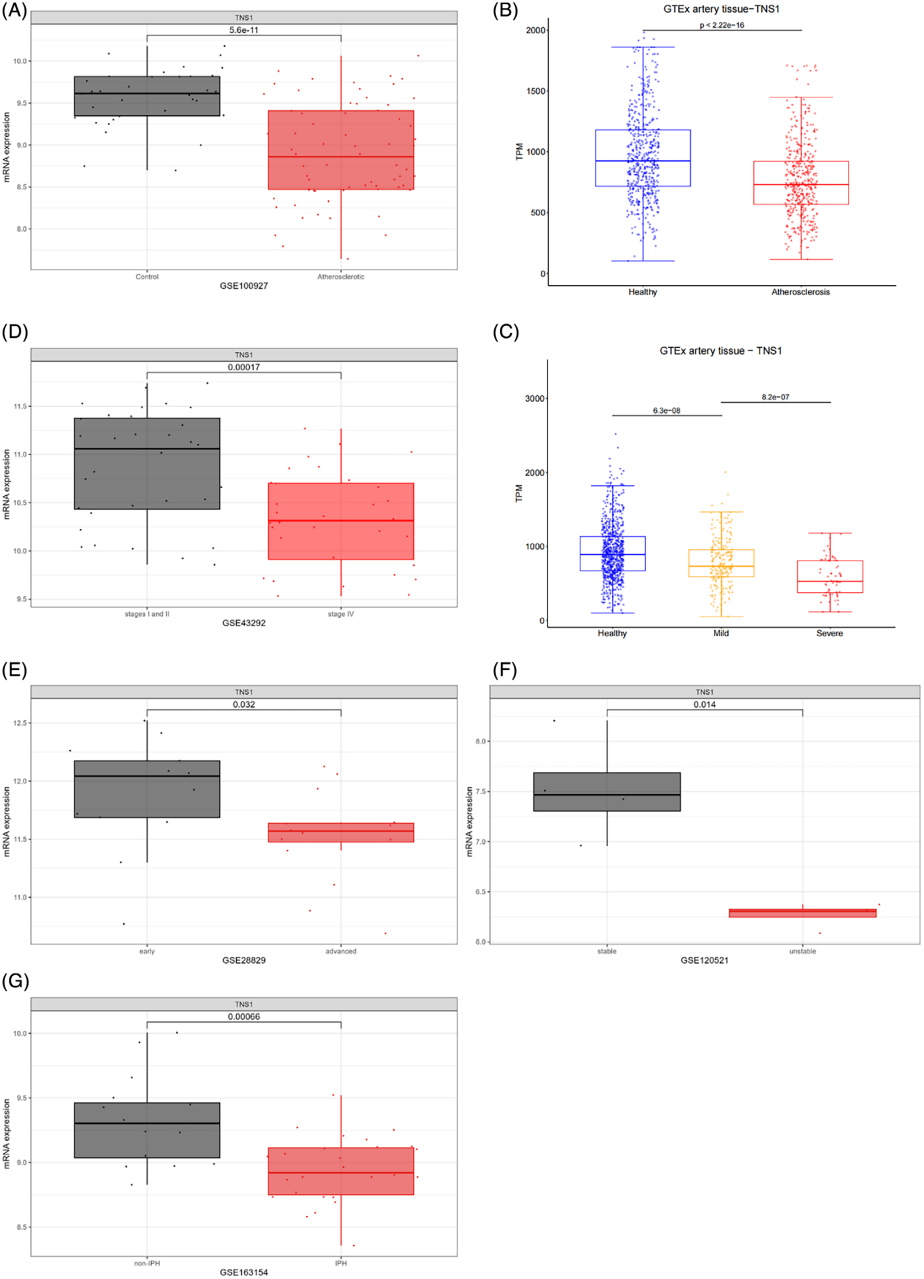

**Figure:**
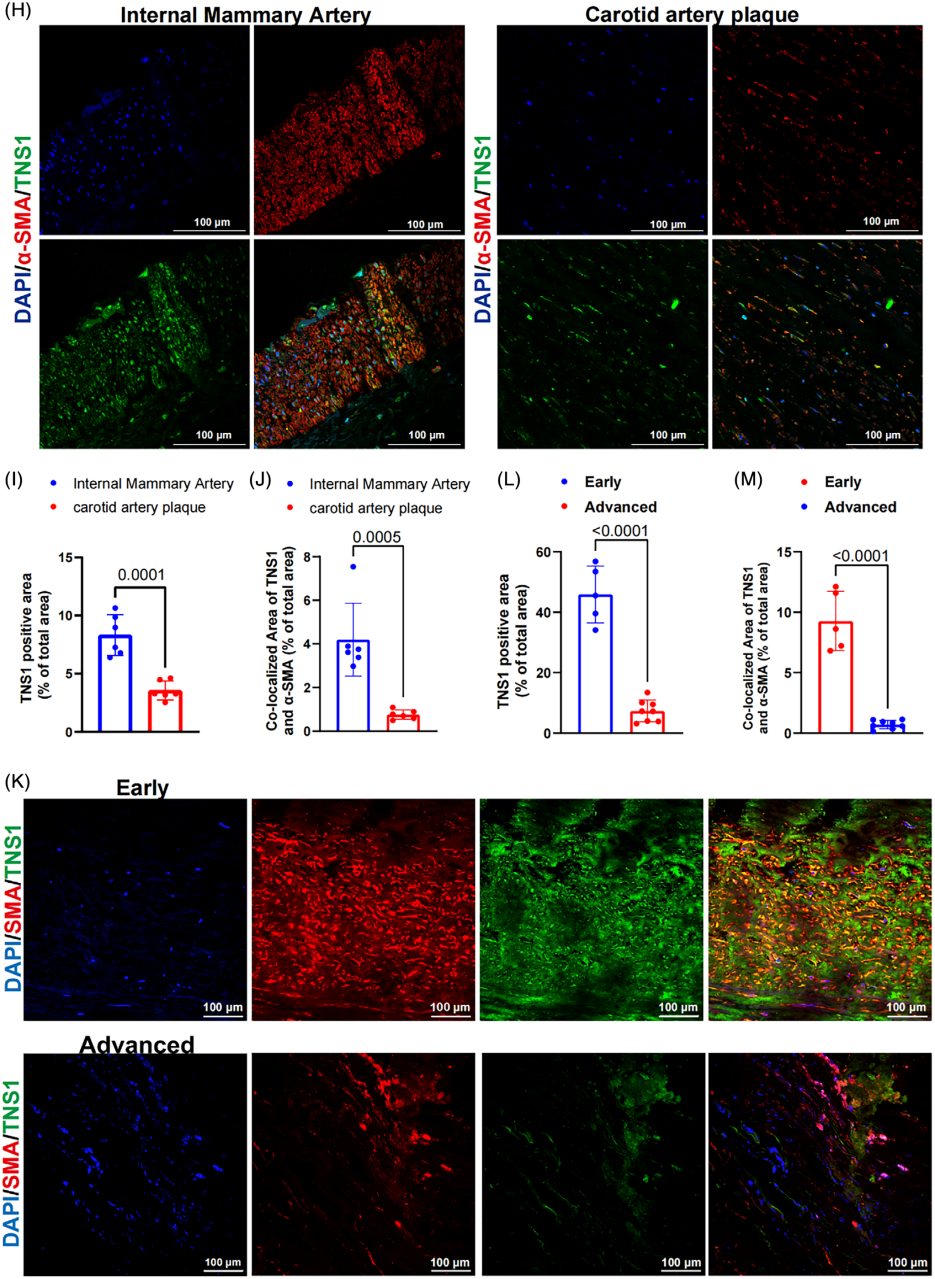

### 3.7 VSMC‐specific *Tns1 gene* deficiency aggravates AS and plaque instability

To validate in vivo role of *Tns1 gene* in AS in the context of VSMCs, we generated *Tns1* ^SMCKO^ApoE*^−/−^* mice through crossing *Tns1* ^flox/flox^ *ApoE^−/−^* mice with Tagln‐Cre mice (Figure S12). Then, *Tns1* ^SMCKO^ *ApoE^−/−^* mice and *Tns1* ^flox/flox^ *ApoE^−/−^* littermates were fed a HFD for 12 or 20 weeks to establish an atherosclerotic model. There was no significant differences between the genotypes in body weight, plasma total cholesterol (TC) and triglycerides (TG; data not shown). ORO staining of the aortas revealed that *Tns1* ^SMCKO^ *ApoE^−/−^* mice showed a significant increase in atherosclerotic lesions in the whole aorta compared with *Tns1* ^flox/flox^ *ApoE^−/−^* mice in the AS model induced for both 12 weeks (Figure 7A,C) and 20 weeks (Figure 7B,D), and the degree of AS lesions in *Tns1* ^SMCKO^ *ApoE^−/−^* mice also significantly increased along with the increase of HFD induction time (Figure 7E). However, no significant differences were observed in lesion area in cross‐sections of aortic roots between the genotypes (Figure 7F,H). We then perform a more detailed analysis of aortic root. Compared with *Tns1* ^flox/flox^ *ApoE^−/−^* mice, *Tns1* ^SMCKO^ *ApoE* ^−/−^ mice showed significantly increased necrotic core size (Figure 7G,I), increased MP infiltration (Figure 7J,L), decreased VSMC content (Figure 7J,K) and decreased collagen deposition (Figure 7N,O) in cross‐sections of aortic roots. Based on the alteration in necrotic core size, MP infiltration, VSMC content and collagen deposition, the atherosclerotic lesions in *Tns1* ^SMCKO^ *ApoE* ^−/−^ mice had a higher vulnerability index than in *Tns1* ^flox/flox^ *ApoE^−/−^* mice (Figure 7Q). *Tns1* ^SMCKO^ *ApoE* ^−/−^ mice also displayed a significant decrease in fibrous cap thickness (Figure 7P). Collectively, a higher vulnerability index and decreased fibrous cap thickness demonstrated that VSMC‐specific *Tns1* gene deletion significantly aggravated plaque instability of *ApoE* ^−/−^ mice. It was worth noting that CD68 protein signals co‐localising with α‐SMA^+^ VSMCs were significantly increased in lesion area in cross‐sections of aortic roots of *Tns1* ^SMCKO^ *ApoE* ^−/−^ mice, compared with *Tns1* ^flox/flox^ *ApoE^−/−^* mice (Figure 7J,M), demonstrating that VSMC‐specific *Tns1* gene deficiency indeed increased MP‐like SMC transdifferentiation.

FIGURE 7Vascular smooth muscle cell (VSMC)‐specific *Tns1* gene deficiency facilitates atherosclerosis and plaque instability of *ApoE* ^−/−^ mice. (A) Representative image for Oil Red O stained enface aorta of *Tns1* ^SMCKO^ *ApoE* ^−/−^ and *Tns1* ^flox/flox^ *ApoE* ^−/−^ mice induced by 12 weeks of high‐fat diet (HFD) feeding (*n* = 7 per group), scale bars, 5 mm. (B) Representative image for Oil Red O stained enface aorta of *Tns1* ^SMCKO^ *ApoE* ^−/−^ mice (*n* = 7) and *Tns1* ^flox/flox^ *ApoE* ^−/−^ mice (*n* = 12) induced by 20 weeks of HFD feeding, scale bars, 5 mm. (C) Quantification of the lipid deposition in plaques of the whole aorta in A (*n* = 7 per group, induced by HFD for 12 weeks). (D) Quantification of the lipid deposition in plaques of the whole aorta in B (*n* = 7 for *Tns1* ^SMCKO^ *ApoE* ^−/−^ group, *n* = 12 for *Tns1* ^flox/flox^ *ApoE* ^−/−^ group, induced by HFD for 20 weeks). (E) Quantification of the lipid deposition in plaques of the whole aorta in A and B (*n* = 7 per group, HFD for 12 weeks versus HFD for 20 weeks). (F) Representative images for aortic roots sections from *Tns1* ^SMCKO^ *ApoE* ^−/−^ mice (*n* = 5) and *Tns1* ^flox/flox^ *ApoE* ^−/−^ mice (*n* = 9) stained with haematoxylin–eosin (H&E). Scale bars, 200 µm. (G) Representative images for necrotic core in aortic roots sections from *Tns1* ^SMCKO^ *ApoE* ^−/−^ mice (*n* = 5) and *Tns1* ^flox/flox^ *ApoE* ^−/−^ mice (*n* = 9) stained with H&E. Scale bars, 200 µm. (H) Quantification of plaque area in the aortic roots in F (*n* = 5 for *Tns1* ^SMCKO^ *ApoE* ^−/−^ mice, *n* = 9 for *Tns1* ^flox/flox^ *ApoE* ^−/−^ mice). (I) Quantification of necrotic core percentage in the aortic roots in H (*n* = 5 for *Tns1* ^SMCKO^ *ApoE* ^−/−^ mice, *n* = 9 for *Tns1* ^flox/flox^ *ApoE* ^−/−^ mice). (J) Immunofluorescence staining of α‐SMA protein (red) and CD68 (green) was performed in aortic root sections from *Tns1* ^SMCKO^ApoE^−/−^ and *Tns1* ^flox/flox^ApoE^−/−^ mice induced by 20 weeks of HFD feeding. Cell nuclei were stained with DAPI (Blue). Scale bars, 200 µm. (K) Quantification of α‐SMA (red) percentage in aortic root plaque area in J (*n* = 5 per group). (L) Quantification of CD68 (green) percentage in aortic root plaque are in J (*n* = 5 per group). (M) Quantification of percentage of positive areas where CD68 (green) and α‐SMA (red) are co‐localised (*n* = 5 per group). (N) Representative images for Masson staining of aortic root sections from *Tns1* ^SMCKO^ *ApoE* ^−/−^ mice (*n* = 5) and *Tns1* ^flox/flox^ *ApoE* ^−/−^ mice (*n* = 5) induced by 20 weeks of HFD feeding. Scale bars, 200 µm. (O) Quantitative assessment of collagen content area in the aortic root in N (*n* = 5 per group). (P) Quantitative assessment of fibrous cap area in the aortic root in N (*n* = 5 per group). (Q) Quantitative assessment of vulnerability plaque index (VPI). VPI was calculated as VPI = (% necrotic core area + % CD68 area)/(% SMA area + % collagen area). Data were expressed as mean ± SEM. The results in C, D, E, H, I, K, L, M, O, P and Q were evaluated by two‐tailed unpaired *t*‐test.

**Figure:**
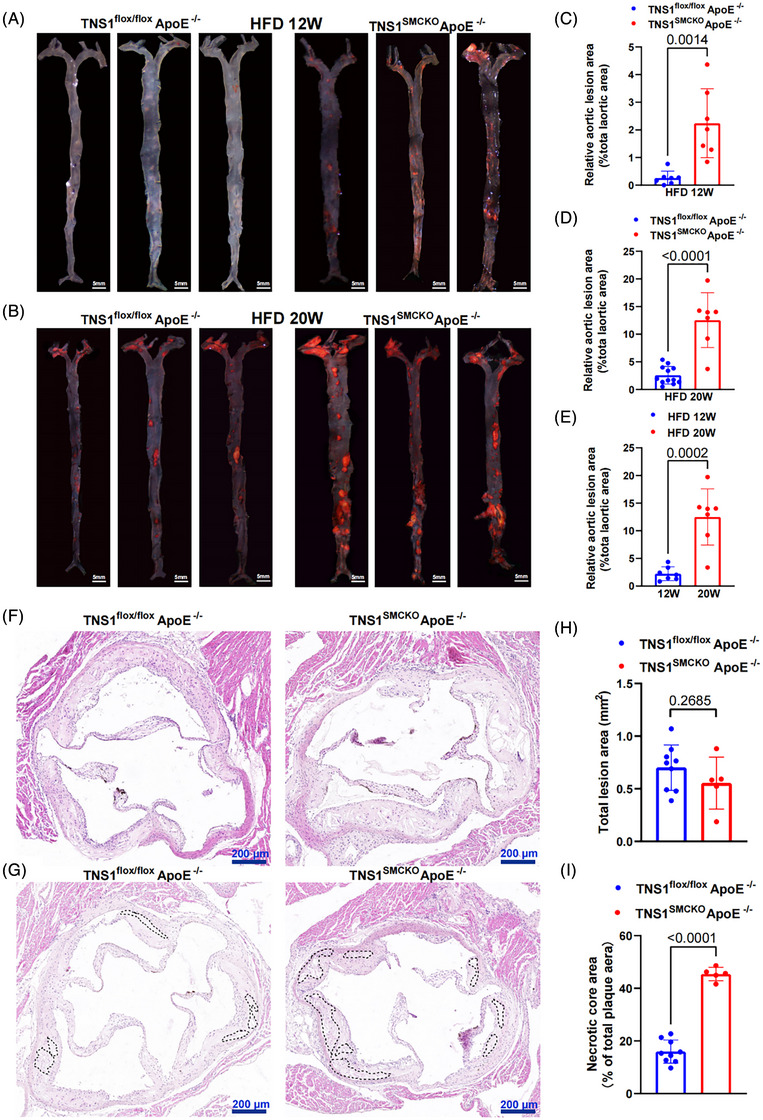

**Figure:**
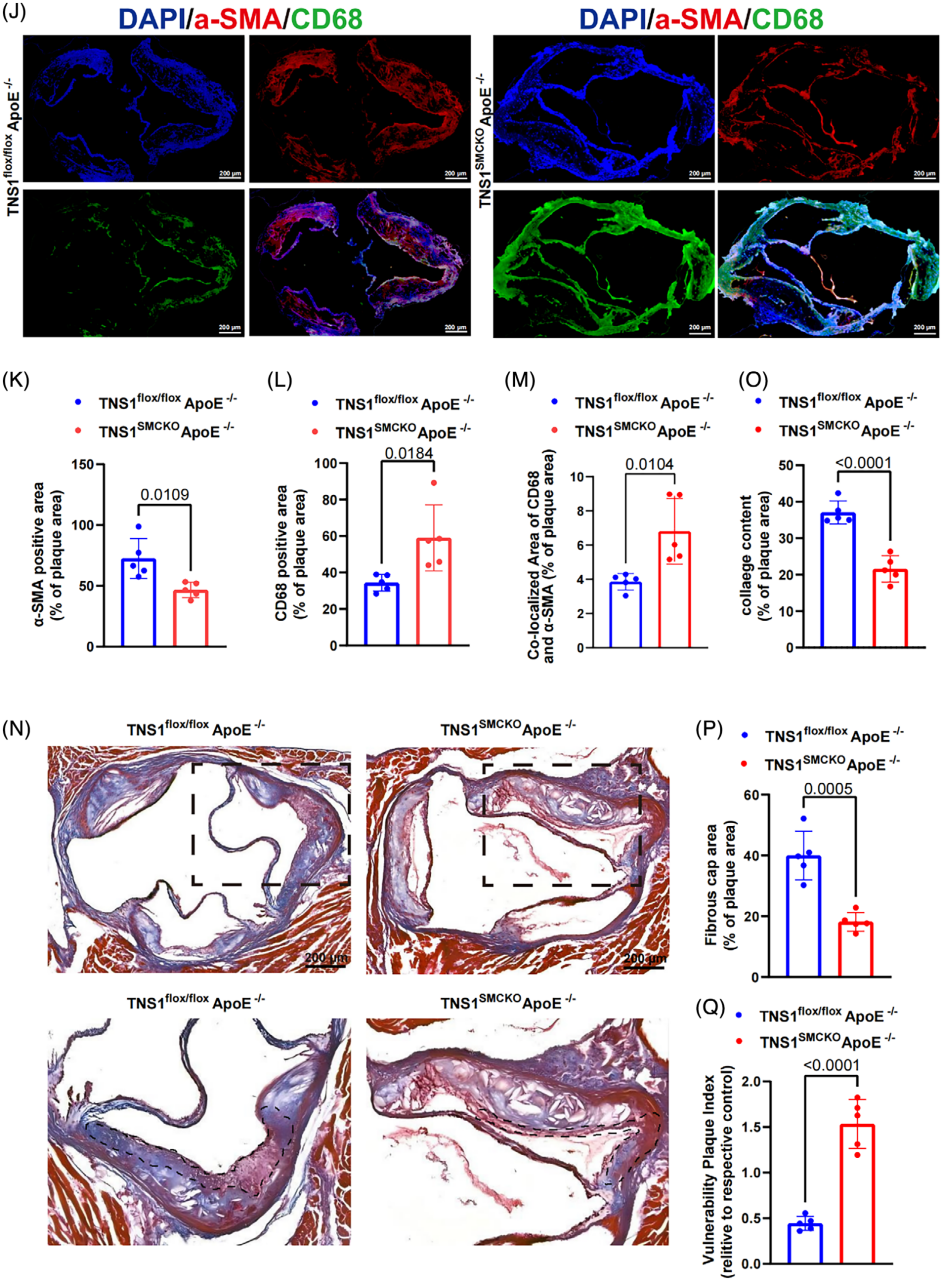

## 4 DISCUSSION

AS represents a major and growing threat to public health.^1^ VSMCs are essential contributors to AS progression and plaque instability.^60^ However, it remains controversial which subtype of VSMCs plays the predominant role in causing the disease.^35^, ^61^ Therefore, this study aims to explore the key VSMC subtype contributing greatly to atherosclerotic plaque instability. To solve this uncertainty, our study performed an integrative analysis of publicly available scRNA‐seq datasets from both SMC‐lineage‐tracing mouse AS models (GSE155513) and unstable human atherosclerotic plaques (GSE253903), identified the MP‐like SMC subtype as the principal contributor to atherosclerotic plaque instability and pinpointed the down‐regulation of *TNS1* gene in VSMCs as the critical event driving this detrimental phenotype switching. VSMC‐specific *Tns1* gene deficiency enhanced contractile VSMC transdifferentiation into MP‐like SMC in vivo, confirming the results of the integrative analysis of scRNA‐seq datasets. Our study shed new light on deeply understanding the controversy in this field: the down‐regulation of *TNS1* gene in VSMCs drived contractile VSMC phenotype switching and subsequent transdifferentiation into MP‐like SMC, ultimately leading to atherosclerotic plaque instability.

We divided VSMCs in scRNA‐seq datasets GSE155513 and GSE253903 into different subtypes based on the known molecular characteristics. The pseudotime trajectory analysis of both scRNA‐seq datasets showed that starting from the contractile VSMC, the MP‐like SMC was at the terminal of the pseudotime stream. The characteristic genes (top 100) in the MP‐like SMC in scRNA‐seq datasets GSE155513 and GSE253903 had significantly higher scores in three independent transcriptome datasets (advanced plaques, unstable plaques and IPH) than in other VSMC subtypes, respectively. These results identified MP‐like SMC as the key SMC subtype contributing the most to atherosclerotic plaque instability. MP‐like SMC exhibited similar expression characteristics in both human and mice, including the up‐regulation of lysosomal and inflammation‐related genes,^9^ which were all enriched in processes such as antigen processing and presentation, leukocyte proliferation, phagosome, lysosomal and regulation of inflammatory responses. In order to identify the hub genes leading to unstable plaques in MP‐like SMC, we performed hdWGCNA analysis, and identified two gene modules in the MP‐like SMCs that were most relevant to the symptomatic patients. These two modules might represent the expression characteristics of MP‐like SMC in unstable atherosclerotic plaques: green module acquired pro‐inflammatory and MP phenotype characteristics and functions, and blue module representing the loss of contractile VSMC phenotype. To be specific, the blue module contained contractile VSMC marker genes and was enriched in processes such as regulating smooth muscle contraction, actin binding and the cytoskeleton of muscle cells. Compared to asymptomatic patients, the expression of these genes in blue module decreased in MP‐like SMCs in symptomatic patients. The green module contained marker genes for MP‐like SMC, such as CD68, CD74 and the HLA family genes (HLA‐DRA, HLA‐DRB1, HLA‐DQA1, HLA‐DQB1, HLA‐DPA1, HLA‐DPB1, HLA‐DMA), which were associated with the functional state of MPs and enriched in processes such as antigen processing and presentation, lysosome, and phagosome. In addition, *AIF1* (Allograft inflammatory Factor‐1) gene, up‐regulated in green module, was reported to promote MP‐like SMC dedifferentiation both in vivo and in vitro.^62^ *RGS5* gene was down‐regulated in blue module, and *CD74* gene was up‐regulated in green module. Interestingly, *Rgs5* ^−/−^ mice showed significant VSMC transdifferentiation into MP‐like SMC,^40^ marked by Cd74^+^Myh11^+^. These results solidly confirmed the accuracy of blue and green modules resulting from hdWGCNA analysis. Therefore, up‐regulation of genes in green module might be responsible for the functions of MP‐like SMC, while down‐regulation of genes in blue module might initiate contractile VSMC phenotype switching and transdifferentiate into MP‐like SMC. Subsequent cross analysis of blue module with differentially expressed genes of MP‐like SMC in scRNA‐seq dataset GSE253903 and LASSO regression analysis in bulk RNA‐seq dataset GSE12052 combined with pseudotime trajectory analysis in scRNA‐seq dataset GSE155513 demonstrated that the decreased expression of Tensin‐1 (*TNS1*) gene in VSMCs might drive contractile VSMC transdifferentiation into MP‐like SMC.

Fblim1‐Tns1‐Synpo2 was recently reported to function as a potential complex correlating with Cytoskeletal Protein Binding and Actin Binding.^21^ *Tns1* gene knockdown in MASMCs led to decreased expression of Myocd gene and contractile VSMC marker genes (*Acta2*, *Cnn1 and Tagln*), elevated expression of *Klf4* gene and enhanced synthetic VSMC phenotype with augmented proliferation ability and greater migration ability of MASMCs.^21^ Thus, TNS1 might act as critical checkpoint protein for maintaining the contractile phenotype of VSMCs. However, it remains unclear which SMC subtype contractile VSMC may transdifferentiate into during pathological remodelling in arterial diseases. In addition, the role of *TNS1* gene in the pathogenesis of AS and atherosclerotic plaque instability remains largely unknown. In the present manuscript, the decreased *TNS1* gene expression in VSMCs was positively correlated with the down‐regulation of contractile VSMC marker genes and negatively correlated with the up‐regulation of *CD68* gene. Accordingly, the expression levels of *KLF4* gene were significantly increased, while the expression levels of *SRF* and *MYCOD* genes were significantly decreased in the *TNS1*‐neg group, compared with the *TNS1*‐pos group. The expression levels of *TNS1* gene was negatively correlated with the pathways involved in extracellular matrix disassembly and cholesterol esterification, indicating that the decreased expression of *TNS1* gene might lead to intensified extracellular matrix disassembly and enhanced cholesterol esterification enabling the storage of free cholesterol in lipid droplets and contributing to MP‐like SMC transdifferentiation. We further performed a multi‐gene Module Score analysis using the Seurat AddModuleScore framework to quantitatively evaluate the functional effects of TNS1 gene expression on the scores of contractile gene signature or inflammatory gene signature in VSMCs. Compared with the *TNS1*‐pos group, the *TNS1*‐neg group demonstrated a dramatic reduction in the contractile score and a significant elevation in the inflammatory score, indicating that the *TNS1*‐neg group represented a biologically distinct, de‐differentiated VSMC state. Subsequent cell communication in the *TNS1*‐neg group was more active than in *TNS1*‐pos group, for example, higher VCAM, PDGF, THBS and CXCL signalling pathways in *TNS1*‐neg group were closely related to AS, smooth muscle phenotype switching and inflammation. These pathways are known to be key regulators of VSMC phenotype switching. These results demonstrated that the decreased expression of *TNS1* gene not only initiated contractile VSMC phenotype switching, but also drived VSMC transdifferentiation into MP‐like SMC probably via down‐regulating *SRF* and *MYOCD* genes and up‐regulating *KLF4* gene in VSMCs. Finally, in vivo experimental results confirmed the role of VSMC‐specific *Tns1* gene deletion in enhancing MP‐like SMC transdifferentiation and atherosclerotic plaque instability, deepening the function of *TNS1* gene in VSMC phenotype switching and AS.

Collectively, these results demonstrated that the decreased expression of *TNS1* gene not only initiated VSMC phenotype switching, but also drived contractile VSMC transdifferentiation into a dedifferentiated, pro‐inflammatory state of MP‐like SMC probably due to the transcriptional imbalance or intensified extracellular matrix disassembly and enhanced cholesterol esterification induced by *TNS1* loss. However, the precise mechanism leading to transcriptional imbalance or dysregulated extracellular matrix disassembly and cholesterol esterification in the present study remains still unknown. Structurally, TNS1 is known to function as a focal adhesion adaptor protein that can interact with the barbed ends of actin filaments and crosslink these filaments and link integrin receptors to the actin cytoskeleton through its N‐terminal actin‐binding domain and C‐terminal phosphotyrosine‐binding domain.^63^ Whether this structural property contributes to reveal the molecular mechanism of TNS1 in driving contractile VSMC transdifferentiation into MP‐like SMC in the context of AS remains to be resolved in the future work. Beyond its structural role, TNS1 actively transduces biomechanical cues into biochemical signals. TNS1 recruits and binds to phosphorylated signalling molecules such as focal adhesion kinase (FAK) and phosphoinositide 3‐kinase (PI3K) at adhesion sites. Moreover, TNS1 has been reported to regulate the RhoA GTPase pathway by interacting with DLC1 (deleted in liver cancer 1), a RhoGAP protein, with TNS1 binding suppressing DLC1's RhoGAP activity and thereby enhancing RhoA activit.^64^ Given that RhoA is a master regulator of actin cytoskeletal organisation and focal adhesion dynamics, this TNS1‐DLC1‐RhoA signalling axis may represent a potential mechanism by which TNS1 can conduct integrin‐mediated mechanical signals into cytoskeletal remodelling. Whether the interaction of TNS1 with integrins and cytoskeletal components or other independent pathways operates in driving VSMC phenotype switching and MP‐like SMC transdifferentiation during AS progression will be investigated in the future work.

Our study also evaluated the clinical correlation of *TNS1* gene with AS. In the public microarray gene expression datasets, *TNS1* mRNA expression was lower in atherosclerotic artery tissues than in normal artery tissues, and even lower in advanced lesions, unstable plaques or IPH than in early‐stage AS lesions, stable plaques or non‐IPH, respectively, indicating that the decreased expression of *TNS1* gene might be involved in AS progression and the final rupture of unstable atherosclerotic plaques. Furthermore, the expression levels of TNS1 protein in VSMCs were lower in human atherosclerotic plaques than in healthy arteries, and even lower in advanced plaques than in early plaques. These observations suggested that down‐regulation of *TNS1* gene in VSMCs might have causative roles in the AS progression and unstable plaques. However, the underlying mechanism of the down‐regulated *TNS1* gene within VSMCs in human atherosclerotic plaques remains unknown. A positive feedback loop between ZEB1 and *TNS1* gene in a epithelial–mesenchymal transition cell model,^59^ the expression pattern of *ZEB1* mRNA in scRNA‐seq datasets of GSE253903 and GSE155513 and in the *TNS1*‐neg VSMCs collectively indicated the potential for the transcriptional regulation of *TNS1* gene by ZEB1 in VSMCs. ZEB1 exhibited the highest the regulatory importance score among all predicted TFs, and was most strongly and positively correlated with *TNS1* expression. ZEB1 was also identified as the node with the strongest connection to *TNS1* gene in a *TNS1*‐centred transcriptional network. These results indicated that ZEB1 might function as a core transcriptional activator of *TNS1* gene. The ZEB1 regulon included *TNS1* gene as one of its top target genes, again with a positive correlation. This reciprocal finding further supported a ZEB1‐*TNS1* regulatory axis in VSMCs. Comparable to KLF4, ZEB1 also functioned as a global regulator in VSMCs, ranking among the top TFs. ZEB1 was identified as one of six candidate TFs using Animal TFDB and CHEA datasets. There were four potential binding sites of ZEB1 in the promoter of *TNS1* gene predicted using JASPAR datasets. Collectively, ZEB1 was identified as transcriptional factor positively regulating *TNS1* gene within VSMCs in mouse or human atherosclerotic plaques. However, the transcriptional regulation of *TNS1* gene by ZEB1 and their roles in contractile VSMC transdifferentiation into MP‐like SMC in AS progression should be validated in future research.

Beyond biological validation, the robustness of single‐cell transcriptomic interpretations heavily relies on the computational strategies employed. The selection of specialised analytical pipelines for dissecting cellular heterogeneity is critical, as a recent benchmarking study has demonstrated that different scRNA‐seq‐based copy number variation (CNV) inference methods exhibit varying performance across different sequencing platforms and biological contexts.^65^ Recognising the impact of algorithmic selection, we implemented a tailored computational strategy for our multi‐species analysis. For the GSE155513 dataset, we utilised the CCA integration method to effectively harmonise cells from different genetic backgrounds (*ApoE* ^−/−^and *Ldlr* ^−/−^) and eliminate technical batch effects, while the human atherosclerotic dataset was analysed independently to preserve species‐specific biological signatures. To further safeguard the reliability of the identified *TNS1*‐associated phenotype transitions and ensure they reflect genuine biological phenomena rather than technical artefacts, we employed a multi‐modal cross‐validation approach. Rather than relying on a single algorithmic output, our conclusions for both species were robustly supported by a combination of high‐confidence marker gene identification, GO functional enrichment analysis and the integration of gold‐standard lineage‐tracing datasets. This triangulation of evidence ensures the methodological stringency and scientific validity of the *TNS1*‐mediated modulation of VSMC phenotype reported in this study.

However, limitations included the lack of detailed mechanistic elucidation. Although the study linked the decreased expression of *TNS1* gene to downstream pathways, such as PDGF and THBS signalling and inflammatory factor expression, it has not yet fully elucidated the specific downstream signalling cascades or direct molecular interactors driving these changes in in vivo or in vitro models. Although *TNS1* mRNA significantly decreased in in vitro model of MP‐like SMC transdifferentiation according to the datasets of GSE181362 and GSE47744, the effects of *TNS1* gene on the expression of contractile VSMC marker genes, MP marker gene and inflammatory factors and lipid deposition should be validated in vitro and in vivo assays. Although VSMC‐specific *Tns1* gene deletion demonstrated an increased MP‐like SMC transdifferentiation, SMC‐lineage‐tracing mouse AS models should be used to elucidate the role of *Tns1* gene deficiency in driving SMC transdifferentiate into MP‐like SMC from the origin of SMC. While in vivo experimental results indicated that VSMC‐specific *Tns1* gene deficiency increased AS plaque instability, its precise involvement in the rupture of unstable atherosclerotic plaques was yet to be elucidated. Unstable plaque models would be employed to explore the role of *Tns1* gene in the rupture of unstable plaques in the future work. These gaps highlight important future directions focused on delineating the complete molecular pathway and establishing TNS1's potential as a clinical biomarker or therapeutic target for atherosclerotic plaque instability.

## 5 CONCLUSIONS

In summary, MP‐like SMC was identified as the major SMC subtype contributing to atherosclerotic plaque instability and VSMC‐specific *Tns1* gene deficiency aggravated atherosclerotic plaque instability and increased MP‐like SMC transdifferentiation, suggesting that targeting *TNS1* gene in VSMCs may be a novel and promising therapeutic strategy for treatment and prevention of AS and plaque instability.

## AUTHOR CONTRIBUTIONS

**Dongfeng Gu and Laiyuan Wang**: conceived and supervised the research. **Shuang Yang, Rui Fu, Xiaoxiao Ren, Mengyi Sun, Zhifan Li and Yongchun Cui**: performed datasets analysis, immunofluorescence staining and animal experiments. **Shuang Yang, Rui Fu and Laiyuan Wang**: draft the manuscript. **Chenyang Shen, Xianqiang Wang, Naqiong Wu, Bin Yang, Na Shi, Jue Ye, Shufeng Chen and Xiangfeng Lu**: reviewed and edited the manuscript.

## FUNDING INFORMATION

This work was supported by the National Natural Science Foundation of China (Nos. 82570540, 82170480 and 82030102), the CAMS Innovation Fund for Medical Sciences (CIFMS) (No. 2021‐I2M‐1‐010 and 2021‐I2M‐1‐008) and National High Level Hospital Clinical Research Funding (2024‐GSP‐TJ‐15).

## CONFLICT OF INTEREST STATEMENT

The authors declare no conflicts of interest.

## ETHICS STATEMENT

The carotid atherosclerotic plaques of patients undergoing carotid endarterectomy and the internal mammary artery specimens were obtained from Fuwai Hospital. The ethical approval of this study protocol was obtained from the Ethics Committee of Fuwai Hospital (No. 2022‐1780).

## CONSENT TO PARTICIPATE

All animal experiments were conducted in accordance with the Guidelines for the Care and Use of Laboratory Animals issued by NIH and were approved by the Institutional Animal Care and Use Committee of Fuwai Hospital (FW‐2022‐0054).

## CONSENT FOR PUBLICATION

The authors have nothing to report.

## Supporting information



SUPPORTING INFORMATION

SUPPORTING INFORMATION

SUPPORTING INFORMATION

## Data Availability

The bioinformatics analyses conducted in this study utilised publicly available datasets obtained from the Gene Expression Omnibus (GEO) repository and Genotype‐Tissue Expression (GTEx) Portal database. The authors confirm that the data supporting the findings of this study are available within the article and its supporting information.
