# Supplementary material for: Re‐analysis of single‐cell transcriptomics reveals a critical role of TNS1 gene in driving contractile VSMC transdifferentiation into macrophage‐like SMC and atherosclerotic plaque instability
Source: Clin Transl Med. 2026 Apr 20;16(4):e70664. doi: 10.1002/ctm2.70664 (PMC13093596; doi:10.1002/ctm2.70664)
Supplement: Supplementary file 1 — SUPPORTING INFORMATION [file CTM2-16-e70664-s003.docx]

**Supplemental Material**

**
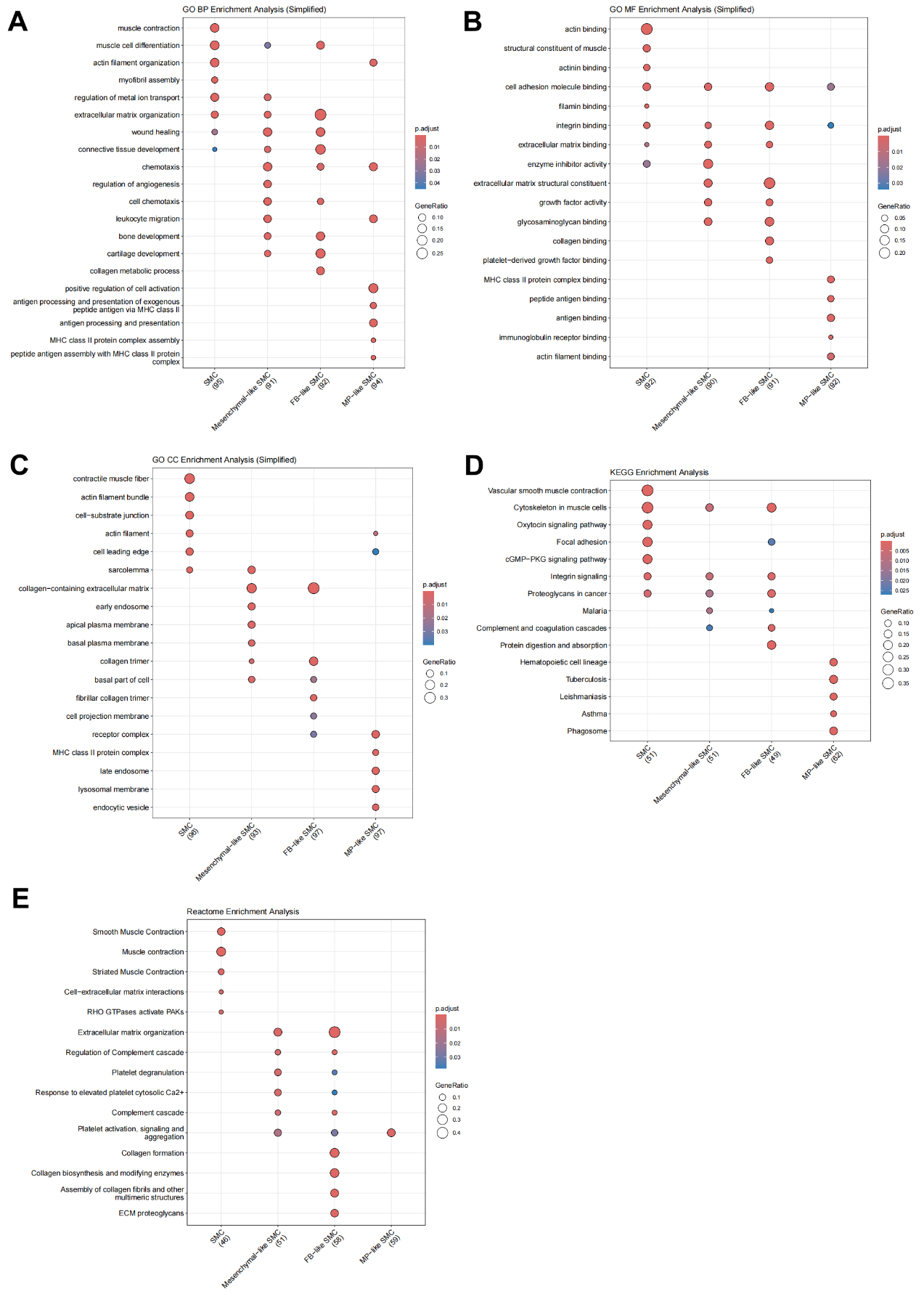
**

**Figure S1. Expression characteristics of SMC** **subtypes** **in scRNA-seq dataset of GSE155513**

**(A-C)** GO analysis results of marker genes in SMC subtypes. The GO analysis includes biological process (BP) (A), cellular component (CC) (B) and molecular function (MF) (C). **(D)** KEGG pathway enrichment results of upregulated genes in SMC subtypes. **(E)** ReactomePA enrichment analysis results of upregulated genes in SMC subtypes.


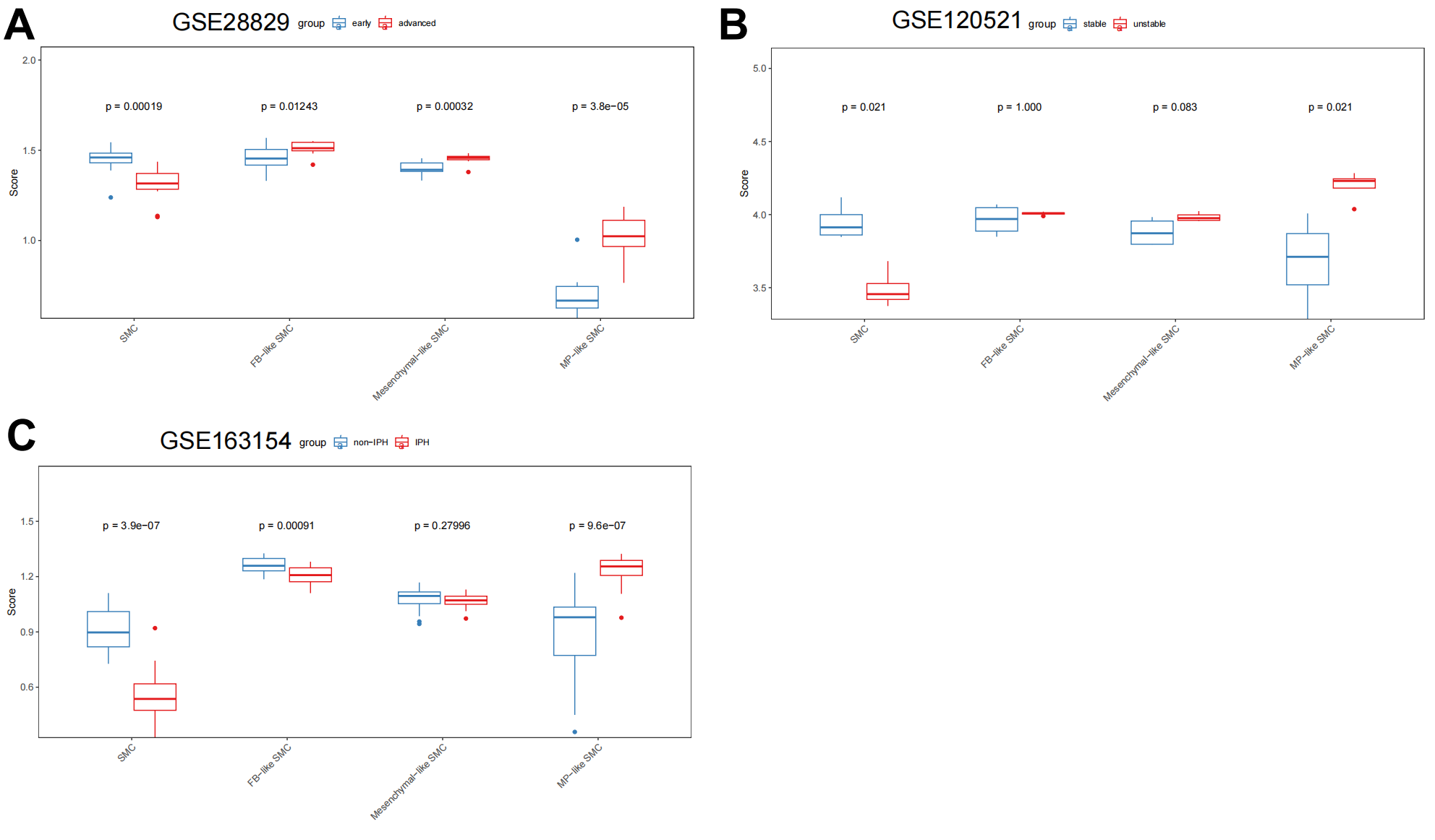


**Figure S2. The signature scores of each SMC subtype (GSE155513)**

**(A-C)** The signature scores of each SMC subtype in bulk transcriptomes of advanced plaques (A), unstable plaques (B) and intraplaque hemorrhaged (IPH) plaques (C). Statistical differences (between the early and advanced groups / stable plaques and unstable plaques /IPH and non-IPH groups) were determined using the unpaired two-tailed Student’s t-test.


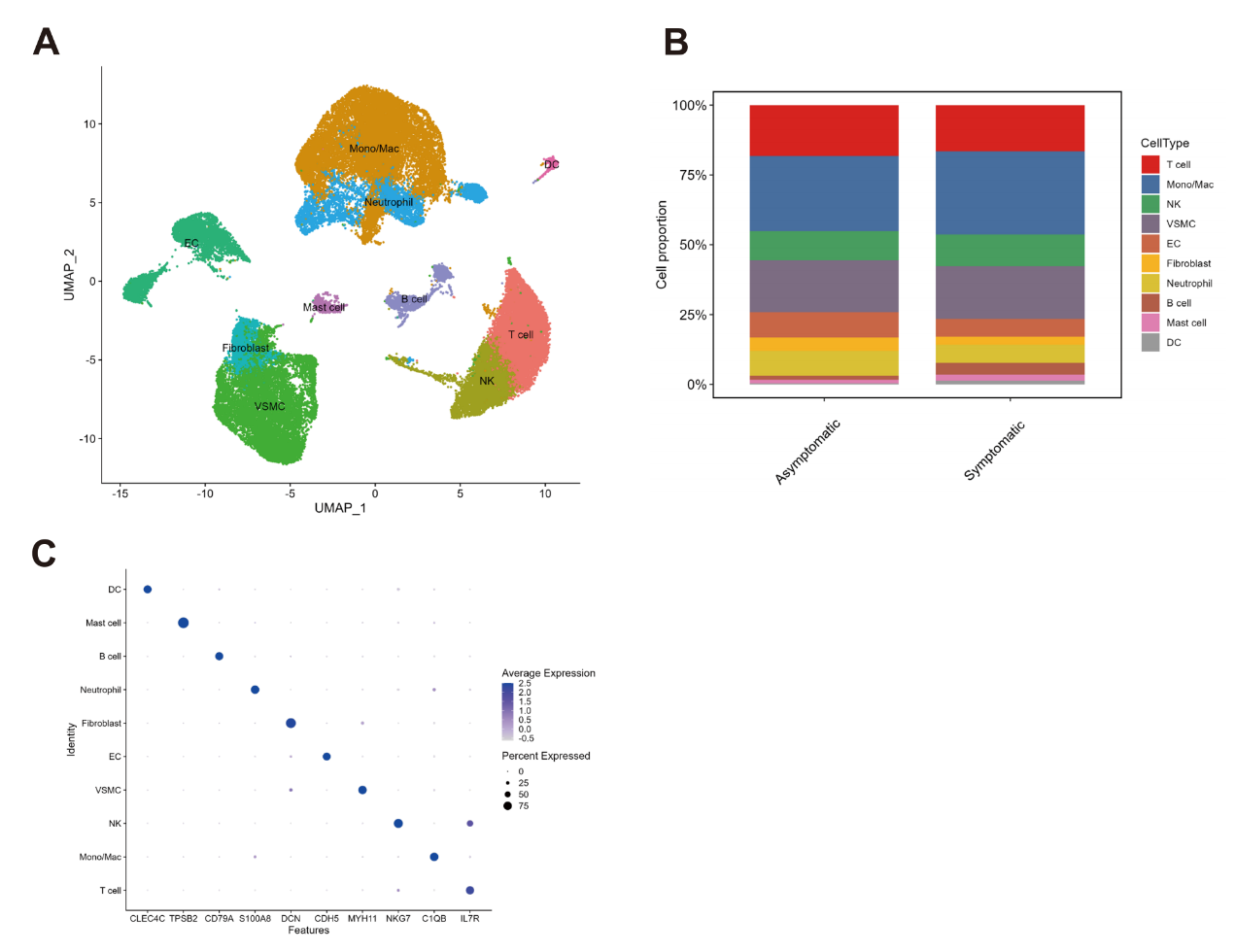


**Figure S3. Cell cluster of GSE253903**

**(A)** UMAP of single-cell RNA sequencing data of human carotid arteries exhibiting cell types. **(B)** Bar chart shows the difference in cell proportions between symptomatic patients and asymptomatic patients. **(C)** Dotplot showing expression of marker genes in each cell types. DC (CLEC4C), B cell (CD79A), VSMC (*MYH11*), endothelial cell (*CHD5*), mononuclear/macrophage (*C1QB*), neutrophil (*S100A8)*, fibroblast (*DCN*), Mast cell (*TPSB2*), natural killer cell (*NKG7*), T cell (*IL7R*).


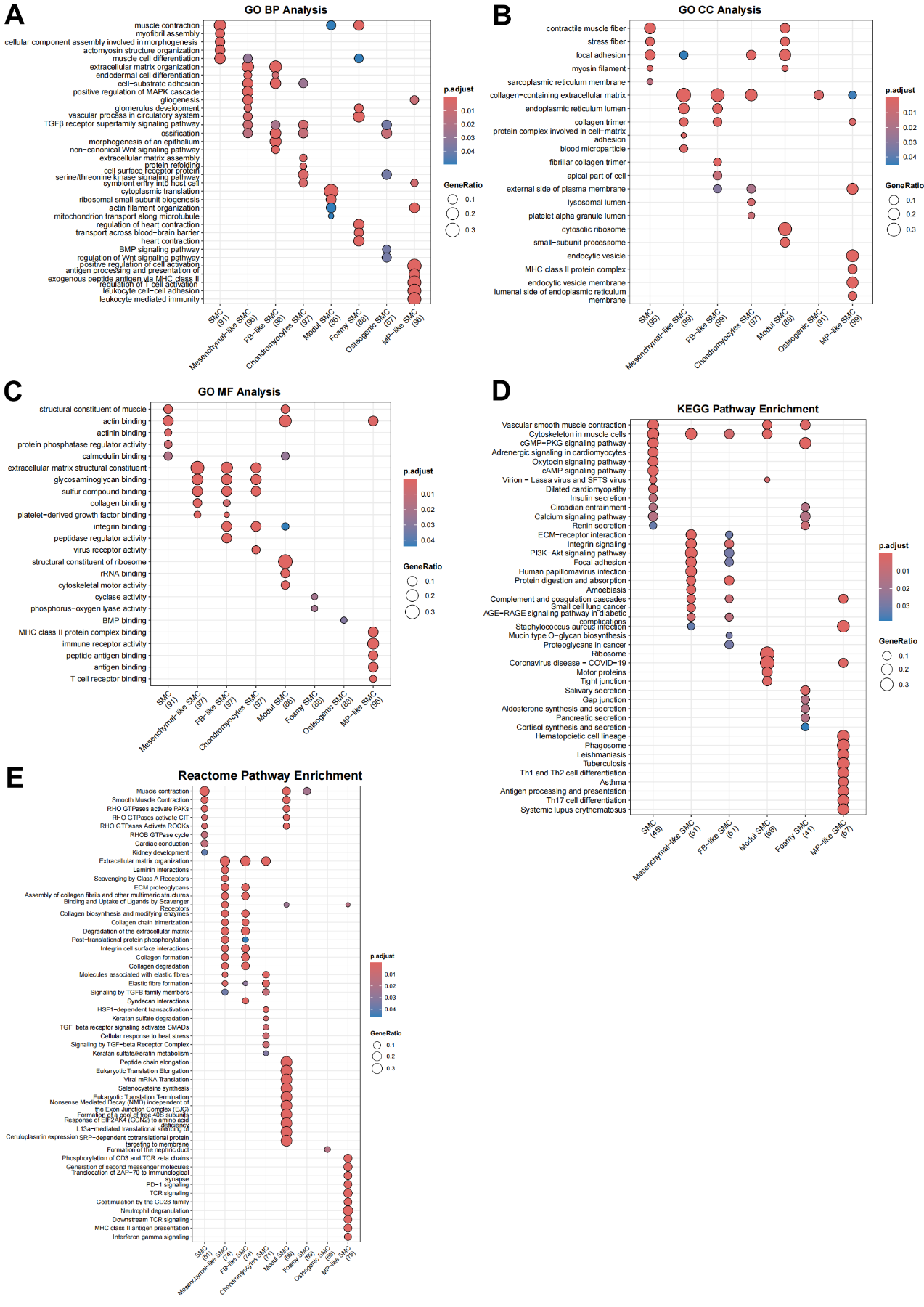


**Figure S4. Expression characteristics of SMC subtypes in scRNA-seq dataset of GSE253903**

**(A-C)** GO analysis results of marker genes in SMCs. The GO analysis includes biological process (BP) (A), cellular component (CC) (B) and molecular function (MF) (C). **(D)** KEGG pathway enrichment results of upregulated genes in SMC subtypes. **(E)** ReactomePA enrichment analysis results of upregulated genes in SMC subtypes.


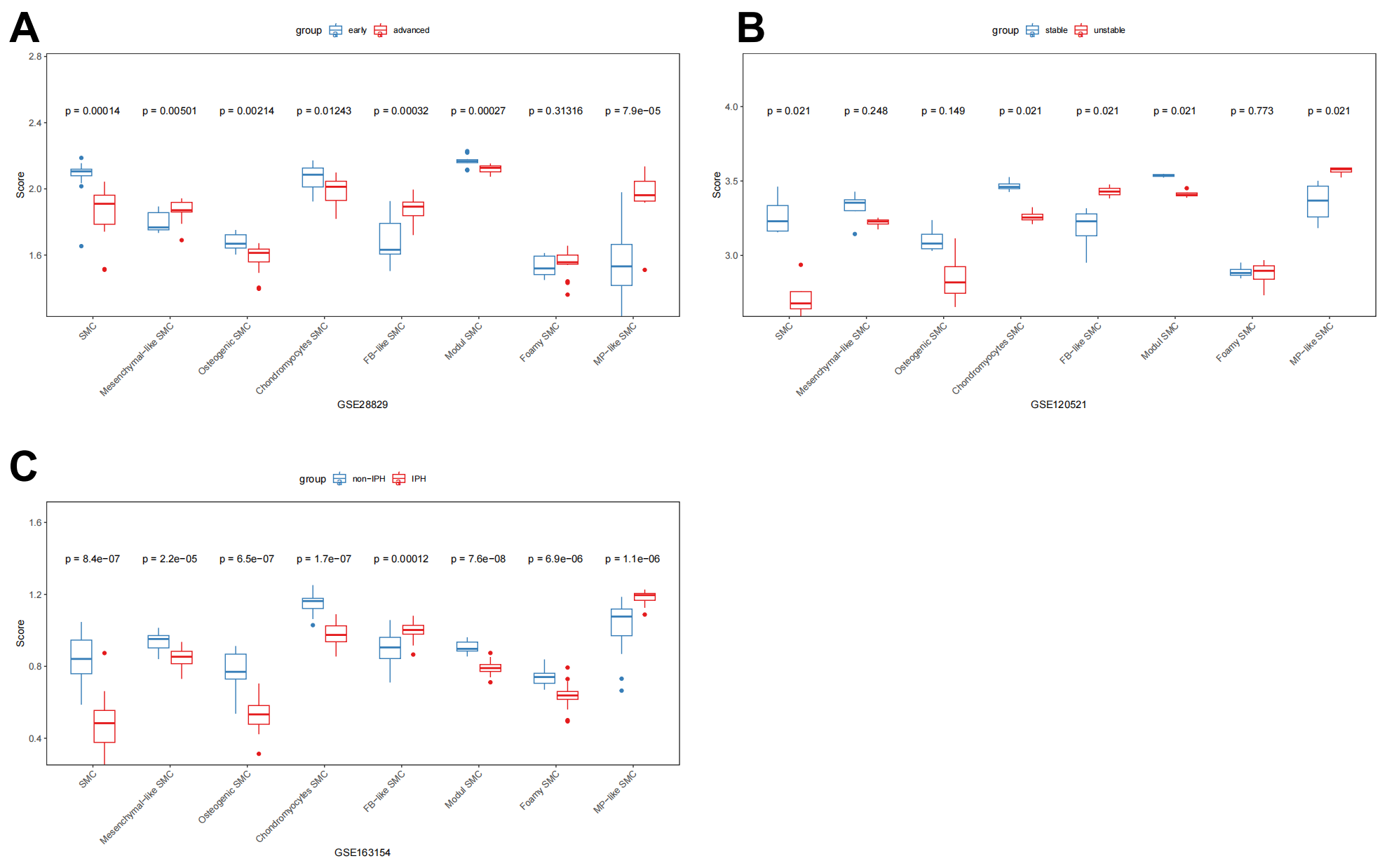


**Figure S5. The signature scores of each SMC subtype (GSE253903)**

**(A-C)** The signature scores of each SMC subtype in bulk transcriptomes of advanced plaques (A), unstable plaques (B) and intraplaque hemorrhaged (IPH) plaques (C). Statistical differences (between the early and advanced groups / stable plaques and unstable plaques /IPH and non-IPH groups) were determined using the unpaired two-tailed Student’s t-test.


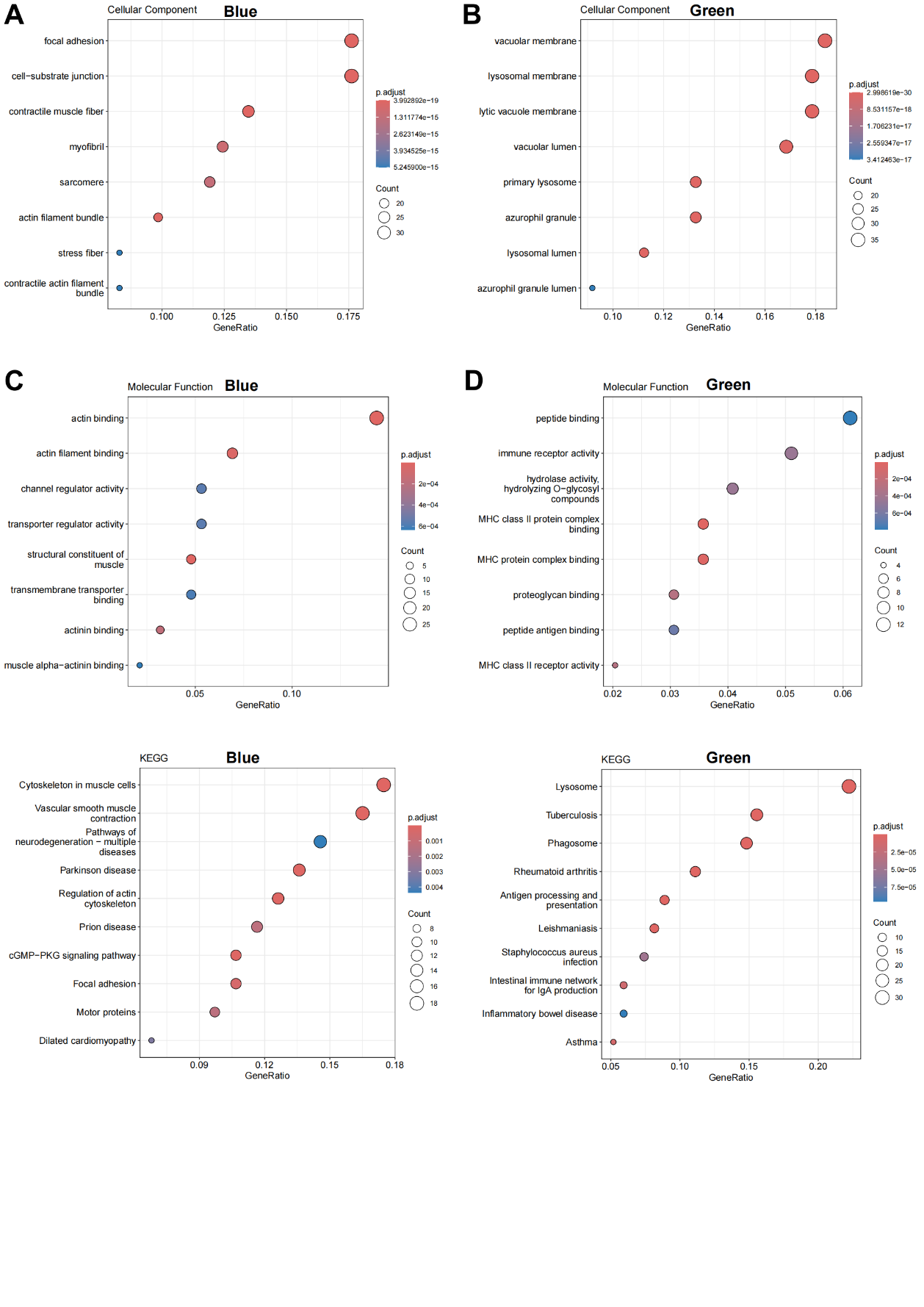


**Figure S6. The expression characteristics of the blue and green modules**

**(A)** GO analysis (CC) results of top100 genes in blue module. **(B)** GO analysis (CC) results of top100 genes in green module. **(C)** GO analysis (MF) results of top100 genes in blue module. **(D)** GO analysis (MF) results of top100 genes in green module. **(E)** KEGG pathway enrichment results of top100 genes in blue module. **(F)** KEGG pathway enrichment results of top100 genes in green module.


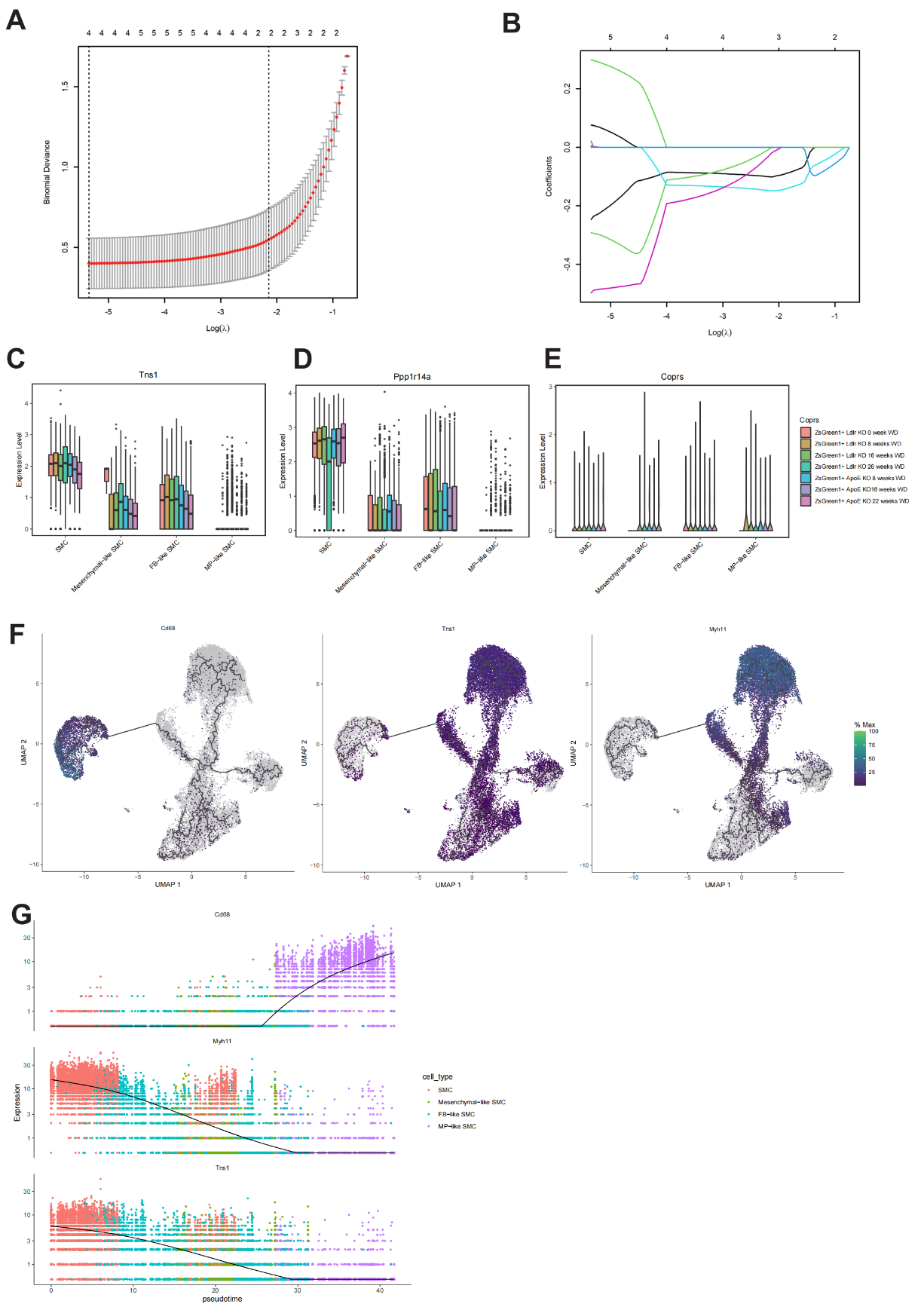


**Figure S7. The expression of the hub genes selected by LASSO**

**(A-B)** hub genes selection by LASSO algorithm. **(C)** *Tns1* expression level in GSE155513. **(D)** *Ppp1r4a1* expression level in GSE155513. **(E)** *Coprs* expression level in GSE155513. **(F)** Pseudotime analysis showing the expression patterns of *Tns1, Myh11 and Cd68* in GSE155513. **(G)** Dynamic transcriptomic shifts along the VSMC-to-macrophage (MP)-like SMC trajectory. The x-axis represents the inferred pseudotime, and the y-axis represents the log-transformed normalized expression levels. Each dot represents a single cell, colored by its identified cell type. Solid lines represent the locally weighted scatterplot smoothing (LOESS) fit for each gene. A clear molecular continuum is observed, characterized by the progressive downregulation of *Myh11* and *Tns1*, which synchronized with the gradual induction of macrophage marker *Cd68*.


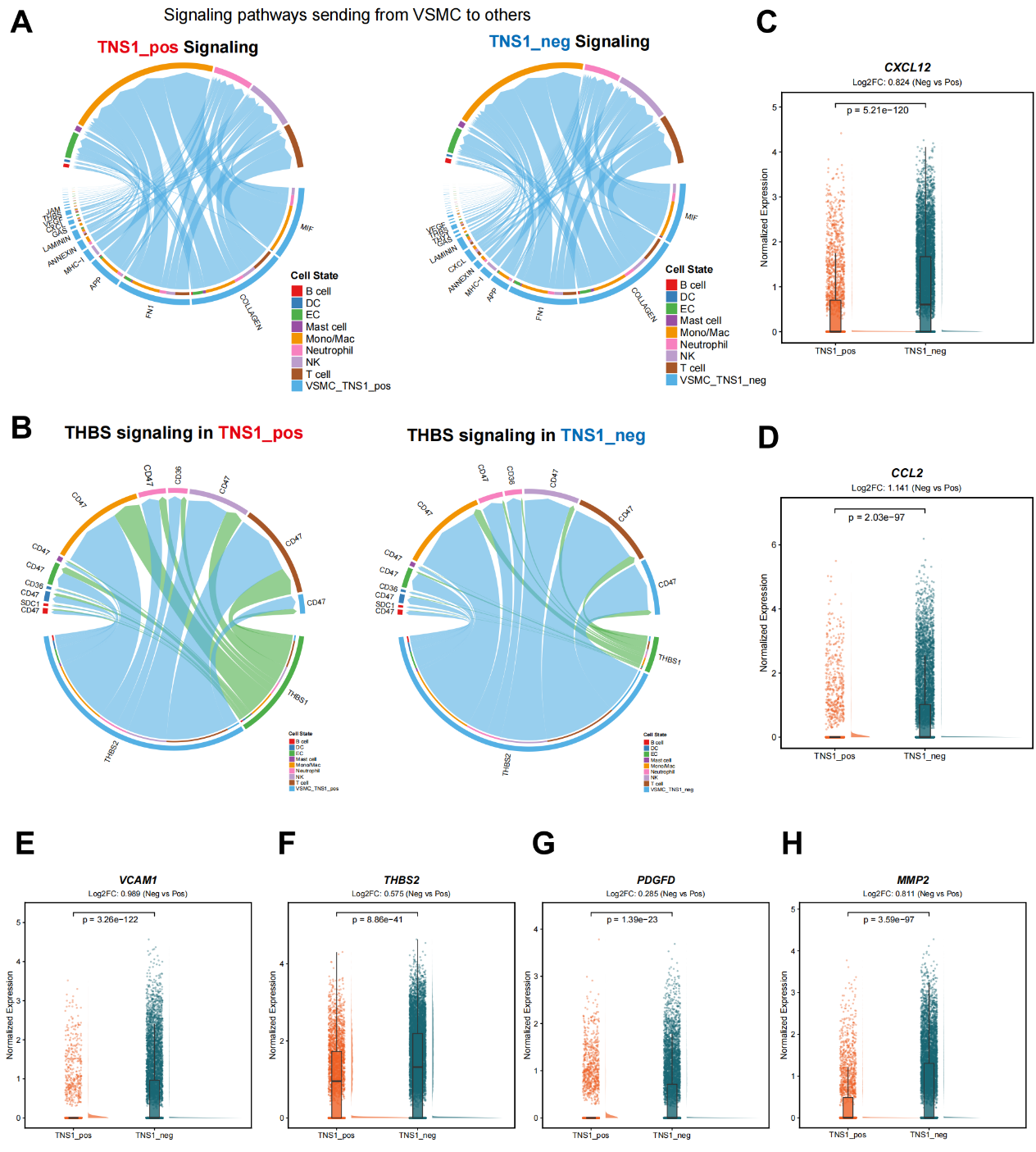


**Figure S8. Cell communication alterations and associated gene expression levels in the *TNS1*-negative group within VSMCs**

**(A)** Chord plots showing the network of signal flows emanating from VSMCs as ligand cells in *TNS1-*pos and *TNS1*-neg groups. **(B)** Chord diagrams showing the ligand-receptor pairs of THBS signaling pathways *TNS1*-pos and *TNS1*-neg groups. **(C-H)** Raincloud plots showing the expression levels of genes (*CXCL12, CCL2, VCAM1, THBS2, PDGFD* and *MMP2*) in *TNS1*-pos and *TNS1*-neg groups. The half-violin plots represent the probability density of gene expression, highlighting the multimodal distribution across cells. Each dot represents an individual cell, with jittering applied to minimize overlap. Central boxplots indicate the median, interquartile range (IQR), and 95% confidence intervals of the expression distribution. log2FC values were indicated in the subtitles. P values were calculated using the Wilcoxon Rank Sum test.


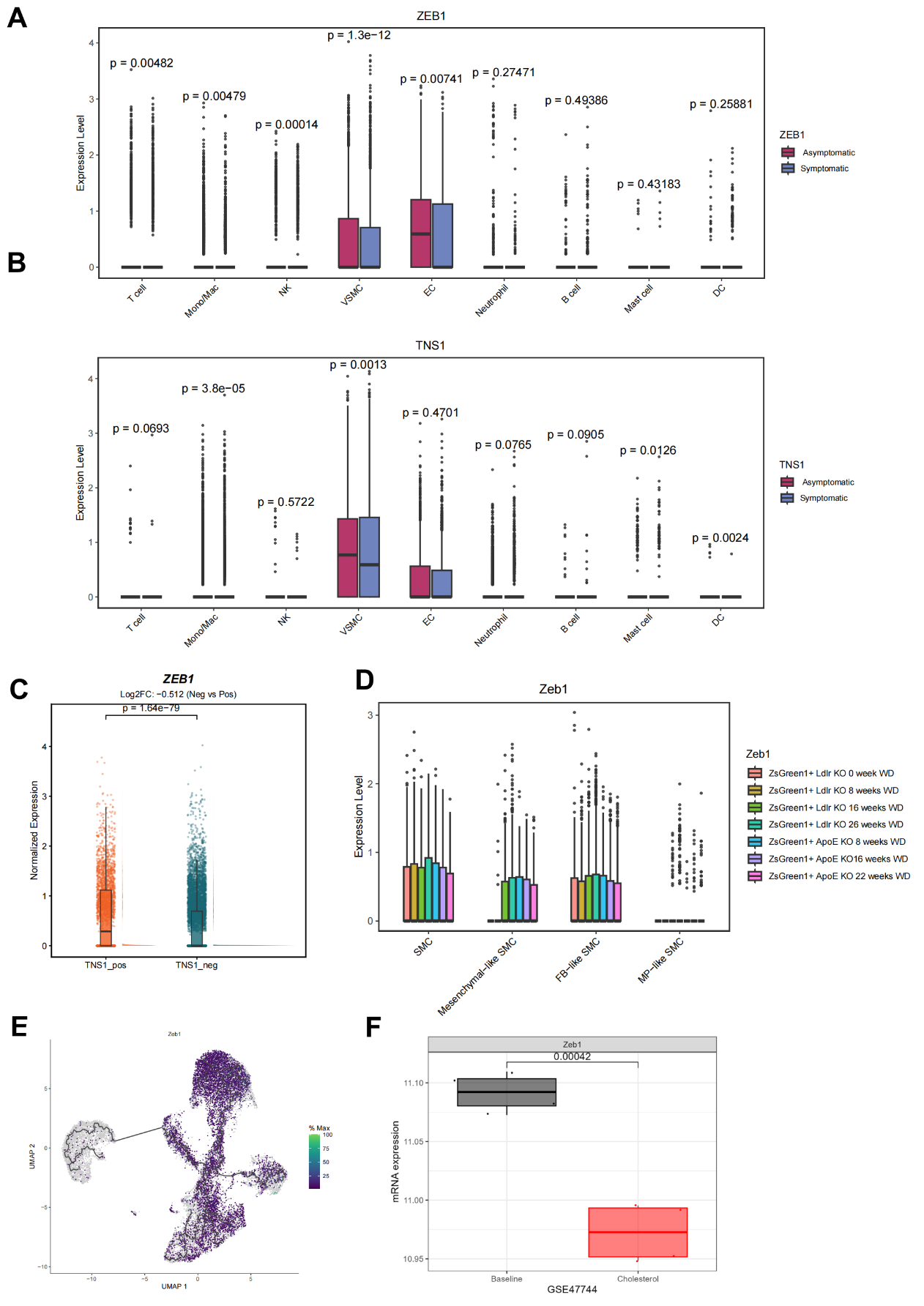


**Figure S9. The expression pattern of *ZEB1* and *TNS1* genes**

**(A)** Boxplots representing the expression levels of *ZEB1* gene in all cells of GSE253903. To account for the typical non-normal distribution of scRNA-seq data, statistical significance between groups within each cell type was determined using the Wilcoxon rank-sum test (via the ggpubr package). Exact P values were labeled above each comparison. (B) Boxplots representing the expression levels of *TNS1* gene in all cells of GSE253903. To account for the typical non-normal distribution of scRNA-seq data, statistical significance between groups within each cell type was determined using the Wilcoxon rank-sum test (via the ggpubr package). Exact P values were labeled above each comparison. **(C)** Raincloud plots showing the expression levels of *Zeb1* gene in *TNS1*-pos and *TNS1*-neg groups. The half-violin plots represent the probability density of gene expression, highlighting the multimodal distribution across cells. Each dot represents an individual cell, with jittering applied to minimize overlap. Central boxplots indicate the median, interquartile range (IQR), and 95% confidence intervals of the expression distribution. log2FC values are indicated in the subtitles. P values were calculated using the Wilcoxon Rank Sum test. **(D)** *Zeb1* gene expression level in SMC subtypes of GSE155513. **(E)** Pseudotime analysis showing the expression patterns of *Zeb1* gene in GSE155513. **(F)** Reanalysis of *Zeb1* gene expression in microarray datasets (GSE447744).


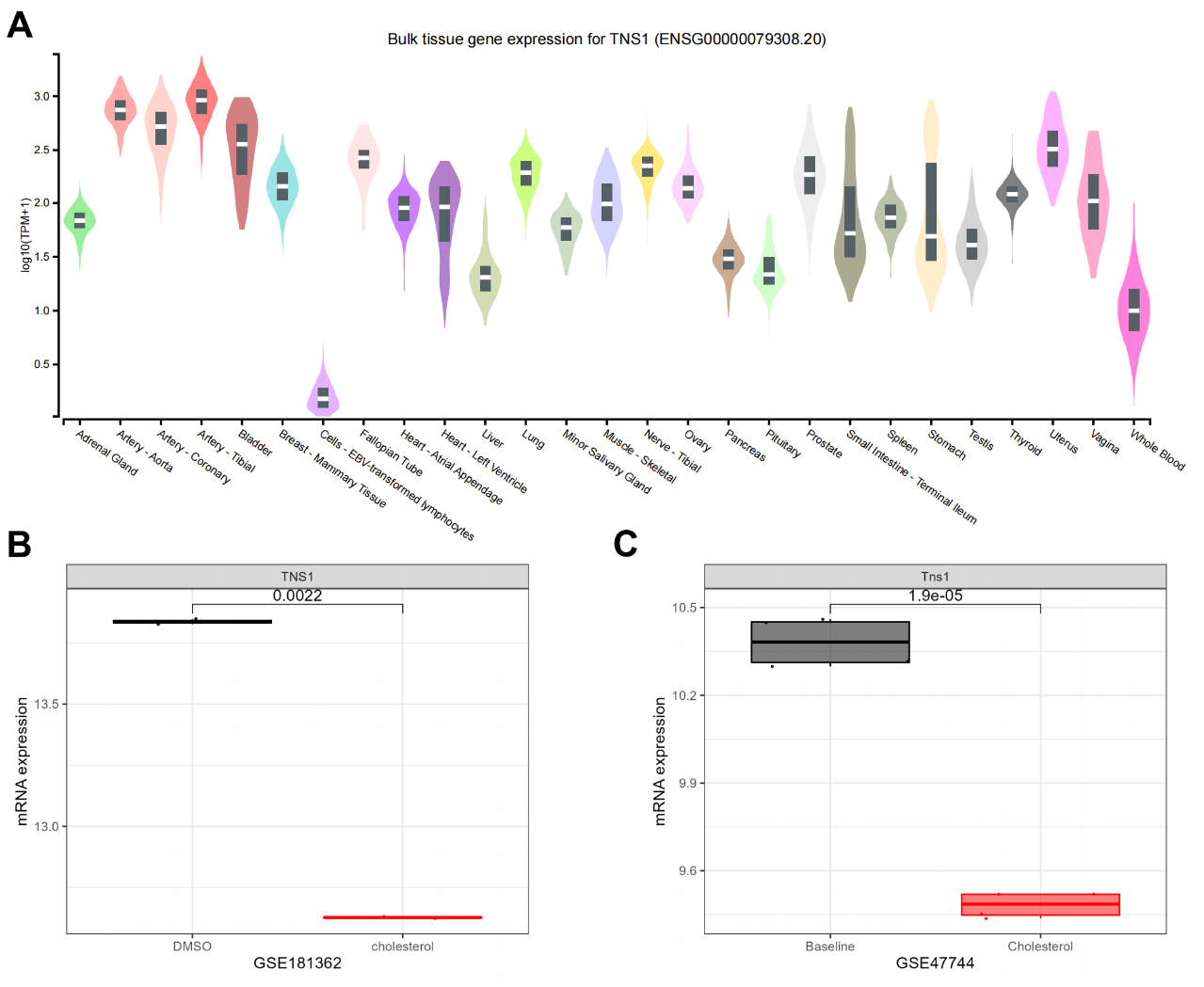


**Figure S10. *TNS1* gene expression across different human tissues (GTEx database) and in the pathology cell model (GEO database)**

**(A)** *TNS1* gene expression across different human tissues**.** The image was created using the GTEx database. **(B)** Reanalysis of *TNS1* expression in RNA-seq datasets (GSE181362). Each dot represents an individual sample. Data were expressed as medians and interquartile ranges. Statistical significance was calculated using two-tailed unpaired t-test. **(C)** Reanalysis of *Tns1* expression in microarray datasets (GSE47744). Each dot represents an individual sample. Data were expressed as medians and interquartile ranges. Statistical significance was calculated using two-tailed unpaired t-test.

**
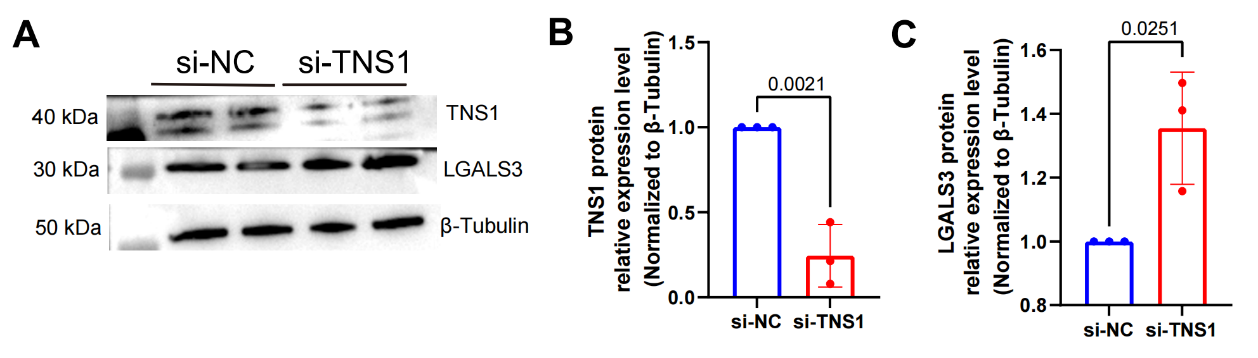
**

**Figure S11. TNS1 knockdown increases LGALS3 expression in human aortic smooth muscle cells**

**(A)** Representative images showing the protein levels of TNS1 and LGALS3 in human aortic smooth muscle cells (HASMCs) transfected with control siRNA (si-*NC*) and *TNS1*-specific siRNA (si-*TNS1*). β-Tubulin was used as a loading control. **(B)** Quantitative analysis of the relative protein levels of TNS1 normalized to β-Tubulin (n =3 per group). **(C)** Quantitative analysis of the relative protein levels of LGALS3 normalized to β-Tubulin (n =3 per group). Data in B and C were presented as mean ± SEM. Data in B and C were evaluated by a two-tailed unpaired t-test.


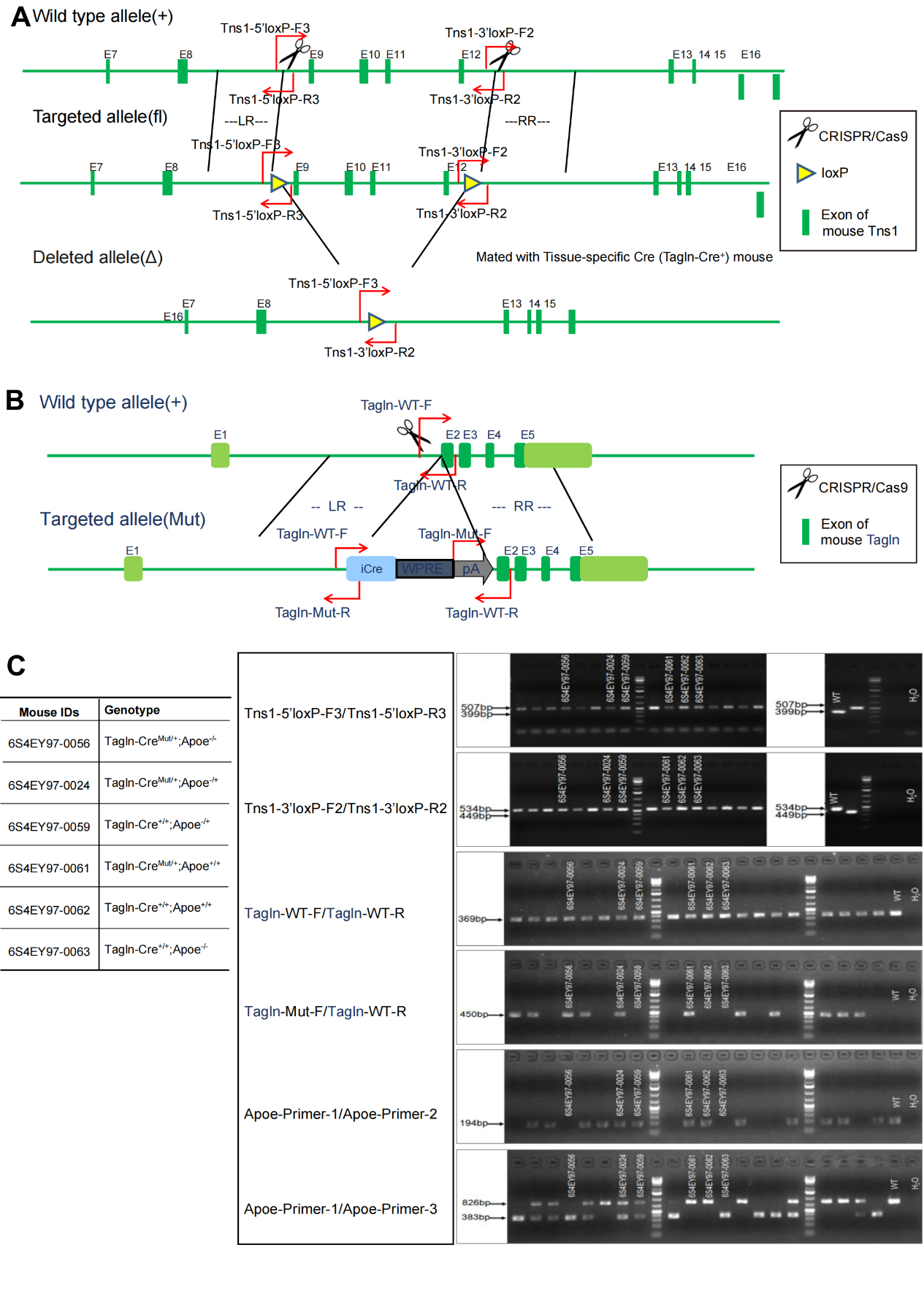


**Figure S12. Generation of VSMC-specific *Tns1* gene knockout mice on the background of** ***ApoE*^−/−^ mice**

**(A),** Targeting strategy and integration detection primer design of *Tns1* flox mice. (**B)**, Targeting strategy and Integration detection primer design of Tagln Cre mice. (**C),** Representative agarose gels showing the results of genotyping of genomic DNA isolated from tails of mice. Primers of *Tns1*-5’loxP-F3/*Tns1*-5’loxP-R3 were used to genotype the 5’loxP: a single 507 bp band indicates homozygosity; two bands at 507 bp and 399 bp indicate heterozygosity; a single 399 bp band corresponds to the wild‑type allele. Primers of *Tns1*-3’loxP-F2/*Tns1*-3’loxP-R2 were used to genotype the 3’loxP: a single 449 bp band indicates homozygosity; two bands at 534 bp and 449 bp indicate heterozygosity; a single 534 bp band corresponds to the wild‑type allele. Primers of Tagln-WT-F/Tagln-WT-R were used to genotype Tagln WT: a single 369 bp band indicates the presence of Tagln WT. Primers of Tagln-Mut-F/Tagln-WT-R were used to genotype the presence of Tagln Mut: a single 450 bp band indicates the presence of Tagln Mut. Primers of Apoe-Primer-1/Apoe-Primer-2 were used to genotype Apoe WT: a single 194 bp band indicates the presence of Apoe WT. Primers of *Apoe*-Primer-1/*Apoe*-Primer-3 were used to genotype *Apoe* Mutant: a single 826 bp band indicates heterozygosity for *Apoe* WT; a single 383 bp band corresponds to heterozygosity for *Apoe* Mutant; two bands at 826 bp and 383 bp indicate heterozygosity.
