# Supplementary material for: Re‐analysis of single‐cell transcriptomics reveals a critical role of TNS1 gene in driving contractile VSMC transdifferentiation into macrophage‐like SMC and atherosclerotic plaque instability
Source: Clin Transl Med. 2026 Apr 20;16(4):e70664. doi: 10.1002/ctm2.70664 (PMC13093596; doi:10.1002/ctm2.70664)

Si-TNS1-Batch 1

Full unedited gel for Supplementary Figures S11A

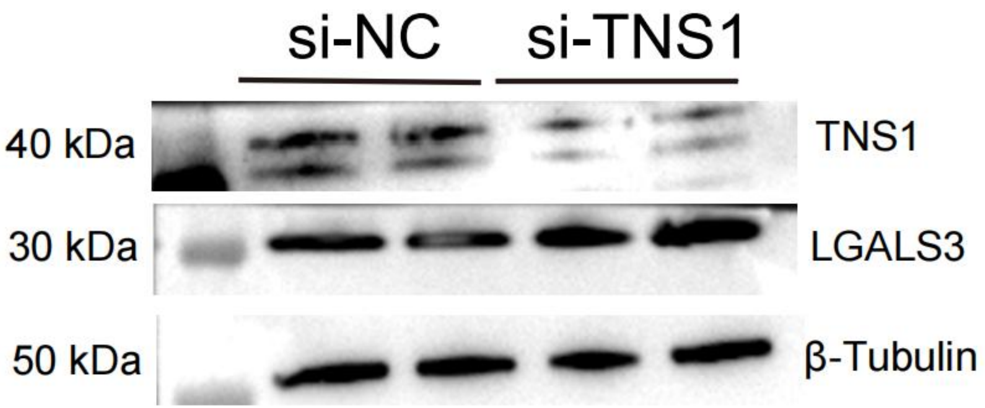

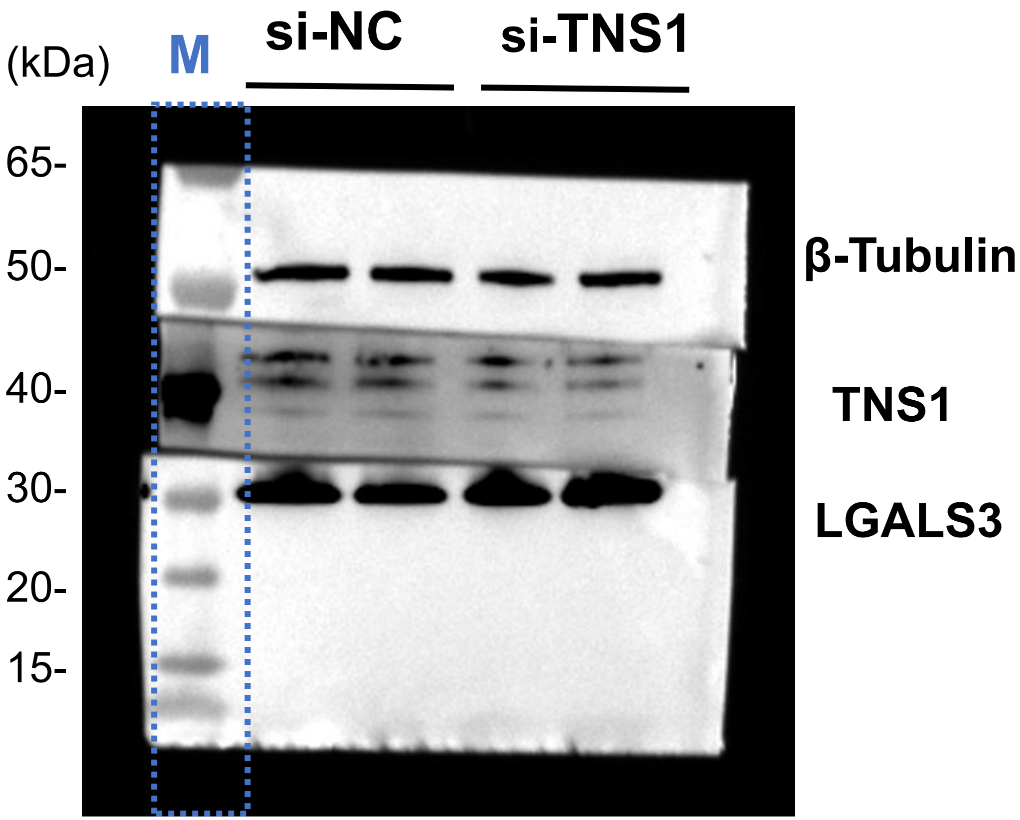

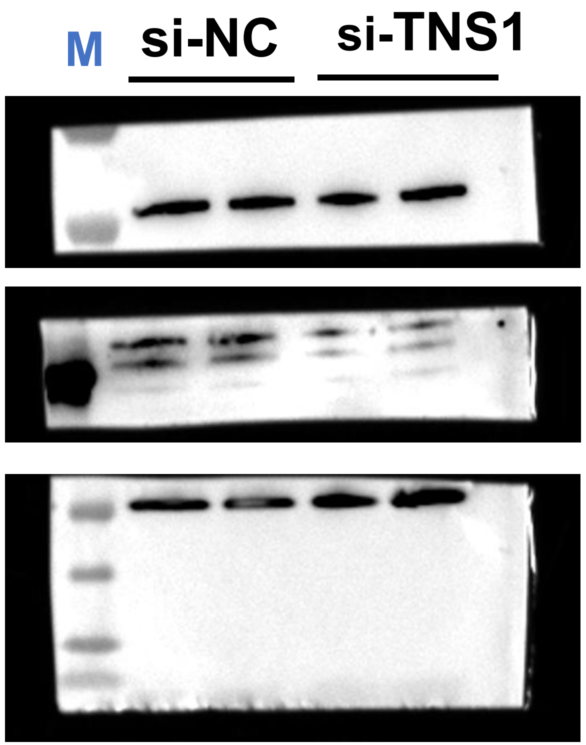

Si-TNS1-Batch 2

Full unedited gel for Supplementary Figures S11A

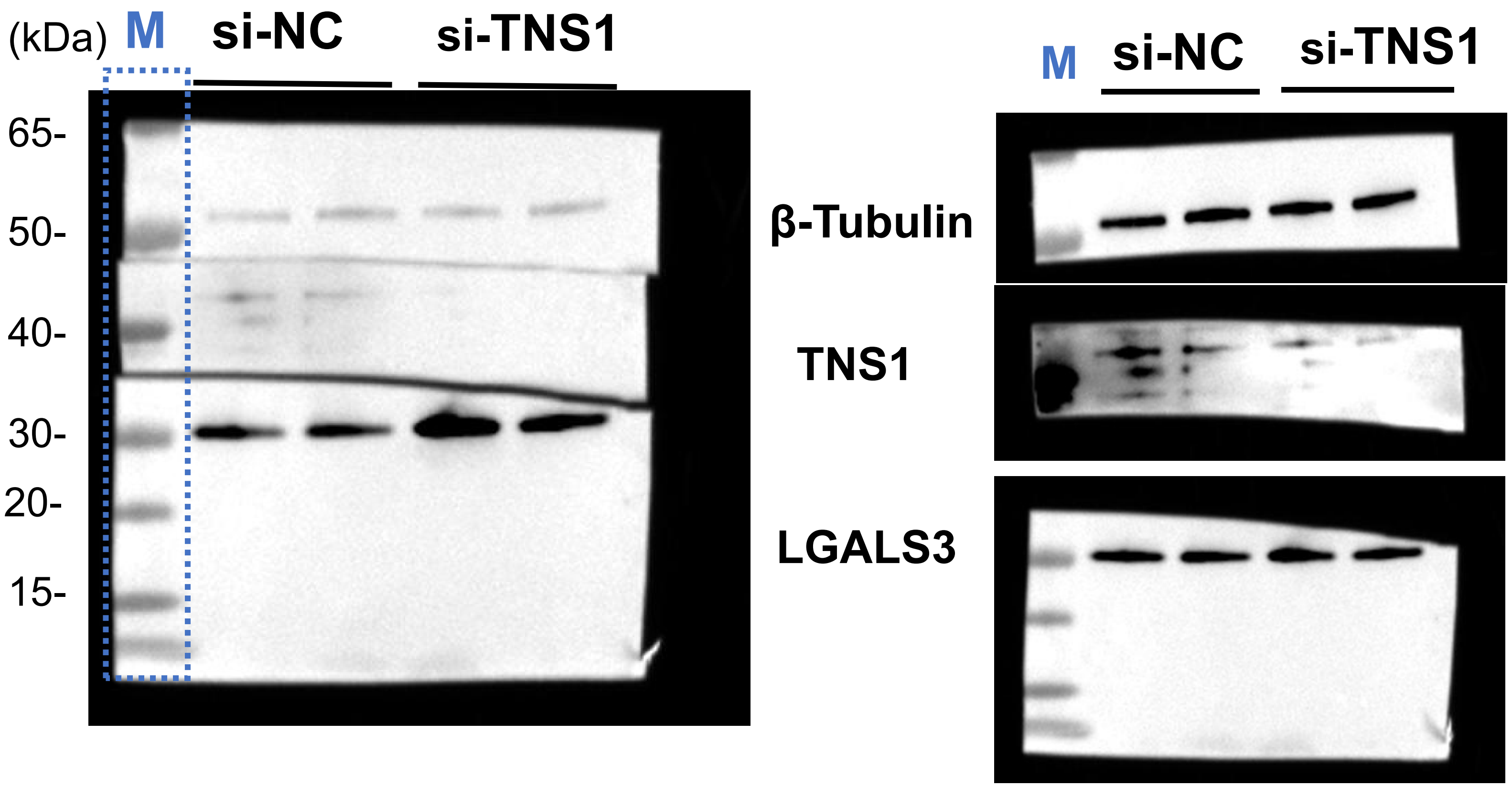

Si-TNS1-Batch 3

Full unedited gel for **Supplementary** Figures S11A

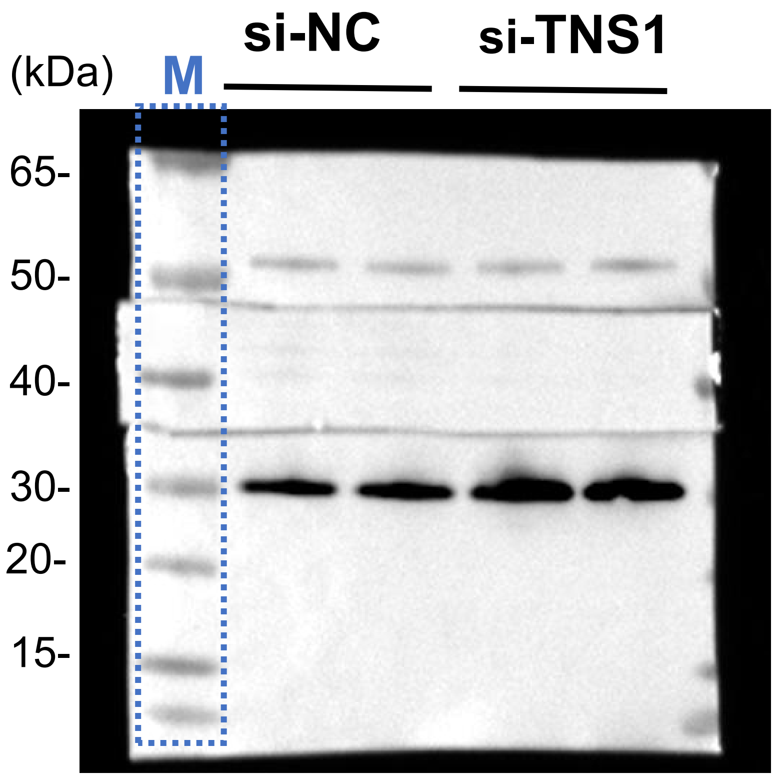

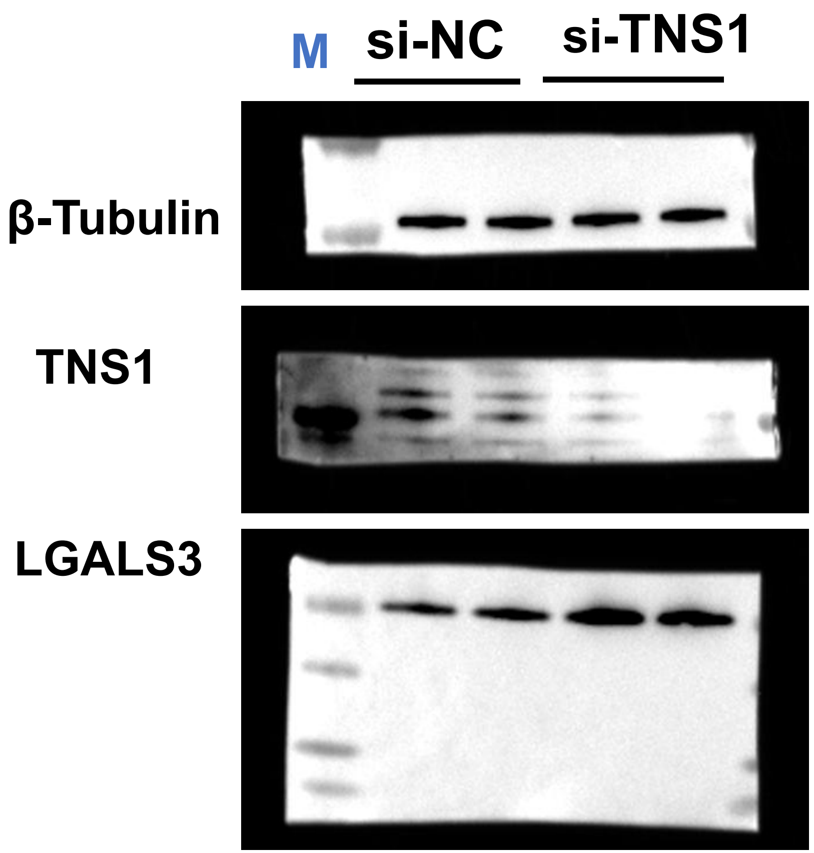

Supplement: Supplementary file 3 — SUPPORTING INFORMATION [file CTM2-16-e70664-s001.pdf]
